# Sensory Navigation System for Indoor Localization and Orientation of Users with Cognitive Disabilities in Daily Tasks and Emergency Situations

**DOI:** 10.3390/s24227154

**Published:** 2024-11-07

**Authors:** María Teresa García-Catalá, Estefanía Martín-Barroso, María Cristina Rodríguez-Sánchez, Marcos Delgado-Álvaro, Robert Novak

**Affiliations:** 1 Department of Computer Science and Statistics, Universidad Rey Juan Carlos, 28933 Madrid, Spain; mt.garciac.2016@alumnos.urjc.es; 2 Department of Applied Mathematics, Materials Science and Engineering, and Electronic Technology, Universidad Rey Juan Carlos, 28933 Madrid, Spain; cristina.rodriguez.sanchez@urjc.es (M.C.R.-S.); marcos.delgadoal@urjc.es (M.D.-Á.); robert.novak@urjc.es (R.N.)

**Keywords:** navigation, location, location and navigation systems, indoor position, sensory navigation, intellectual disabilities, emergency, indoor application

## Abstract

This article presents SmartRoutes, (version 1) a sensory navigation system designed for the localization and guidance of individuals with cognitive disabilities in both indoor and outdoor environments. The platform facilitates route generation in both contexts and provides detailed instructions, enabling effective task execution and seamless integration into daily activities or high-stress situations, such as emergency evacuations. SmartRoutes aims to enhance users’ independence and quality of life by offering comprehensive support for navigation across various settings. The platform is specifically designed to manage routes in both indoor and outdoor environments, targeting individuals with cognitive disabilities that affect orientation and the ability to follow instructions. This solution seeks to improve route learning and navigation, facilitating the completion of routine tasks in work and social contexts. Additionally, in exceptional situations such as emergencies, SmartRoutes ensures that users do not become disoriented or blocked. The application effectively guides users to the most appropriate exit or evacuation point. This combination of route generation and detailed instructions underscores the platform’s commitment to inclusion and accessibility, ultimately contributing to the well-being and autonomy of individuals with cognitive disabilities.

## 1. Introduction

Individuals with cognitive disabilities often face significant challenges in performing everyday tasks, such as navigating different environments and completing routine activities. These difficulties highlight the pressing need for tailored, accessible technological solutions that can support their independence. The ability to move efficiently between locations and carry out daily or work-related tasks is critical for this demographic, yet it remains a significant barrier for many.

Sensory navigation systems have emerged as a promising approach to address these challenges. By integrating localization and guidance technologies, these systems have demonstrated their effectiveness in the public and private sectors, enhancing mobility and safety. Such systems are particularly valuable in supporting cognitive accessibility, where users benefit from customized instructions and route generation adapted to their specific needs.

In recent years, various research efforts have focused on improving these technologies. However, the field still lacks widespread solutions designed specifically for individuals with cognitive impairments in both routine and emergency contexts. This gap highlights the importance of continued innovation to ensure inclusive navigation solutions that empower users and improve their quality of life.

This paper aims to contribute to this field by introducing SmartRoutes Version 1, a sensory navigation system that provides route generation and detailed instructions for users with cognitive disabilities in both indoor and outdoor environments.

The challenges faced by individuals with cognitive disabilities in everyday tasks, such as navigating different spaces and performing routine activities, underscore the critical need for accessible solutions. Tasks like moving between locations or carrying out daily and work-related activities can present significant obstacles for this demographic. In this context, the development of sensory navigation systems plays a crucial role in addressing these challenges and enhancing the independence and quality of life for individuals with cognitive disabilities. These technologies are increasingly common in human services due to their proven effectiveness in both public and private transportation localization and guidance systems. Such systems are key in projects like the European Commission’s connected and automated mobility project [1]. Additionally, localization systems can be found in pre-warning danger devices, such as the well-known V16, which will be mandatory in Spain in 2026 [2].

The V16 device was created by two Spanish entrepreneurs, Jorge Costas and Jorge Torres. Initially, they called it Help Flash, but eventually renamed it V16. This device includes a luminous beacon with yellow flashing lights, which should be placed at the highest point of the vehicle if it is stranded or broken down on the road. The beacon emits signals that cover a 360° field of visibility and enables satellite localization via GPS. This facilitates the identification of the broken-down vehicle by other road users through their geolocation systems, as well as by the DGT (General Directorate of Traffic of Spain) control platform, allowing it to transmit the vehicle’s location in real time and alert other road users about its position through information panels situated along the roads. These devices are battery operated, which gives them a high degree of autonomy.

We can also find outdoor geolocation systems that employ beacons included in wristwatches, such as the Improved Search and Rescue with wrist-worn Personal Locator Beacons, used for rescuing people in danger on boats and/or aircraft that need to be located as quickly as possible [3].

While outdoor localization and positioning systems are quite standardized, relying solely on satellite systems such as GPS III, Galileo (second generation), GLONASS-K, or BeiDou-3 due to their high localization accuracy, indoor localization remains a complex field. Despite the increased attention it has received in recent years, there is still limited literature on research projects addressing indoor localization and positioning systems. This is primarily because locating and positioning people, as well as fixed or mobile objects indoors, requires overcoming a series of challenges dictated by the nature of the environment. Understanding the structure and materials of buildings and indoor spaces where localization takes place is crucial, as these factors can interfere with or distort the propagation of the wave signals used for guidance and localization.

By focusing on both indoor and outdoor navigation, route generation, and providing detailed instructions, these systems aim to offer tailored support for individuals with cognitive disabilities across various environments, including daily routines and emergency situations. This approach underscores a commitment to inclusivity and the empowerment of individuals with cognitive disabilities, emphasizing the importance of creating technology-driven solutions that enhance accessibility and autonomy. This need has opened new research fields to explore technological solutions that can help overcome these challenges and develop tools to assist people in navigating and moving safely through these environments under various circumstances. Current studies employ different technologies for indoor localization and positioning, such as Low Energy Bluetooth (Beacons) [4], magnetic fingerprints [5], Li-Fi Light Fidelity [LED] [6], Wireless Local Area Network [WLAN or Wi-Fi], Wi-Fi fingerprinting [7], ultrasonic signals [8], and acoustic signals [9]. Their uses can be multiple, including solutions for guidance and information in museums, guiding and product sales in shopping centers, warehouse management, passenger guidance in airports or train stations, guidance in hospitals and in large buildings of all kinds, etc.

**Figure 1:**
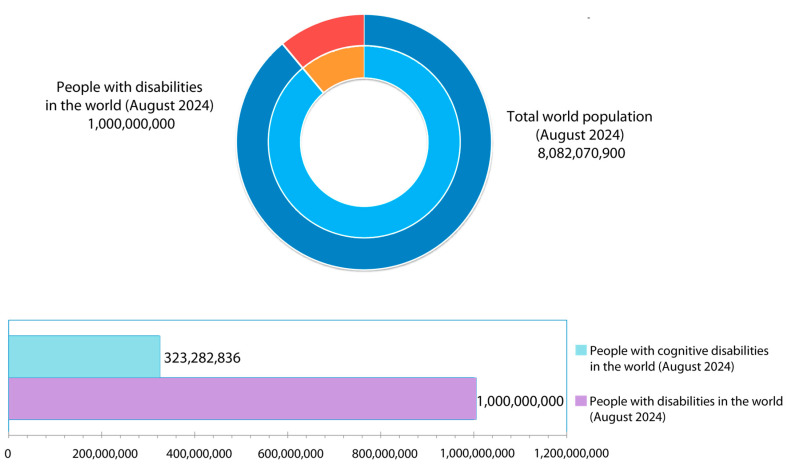
World population with disabilities.

The goal of this research is to provide a tool for indoor localization to effectively locate and position people in their daily activities and in high stress situations, such as emergency evacuations. Furthermore, the tool must be accessible for users with mild to moderate cognitive disabilities. According to the latest 2023 reports from the United Nations [10], it is estimated that there are 1 billion people worldwide, or 1 in 6 people, who have a disability. Of these, it is estimated that between 1% and 4% of the global population [11], or approximately between 80,820,709 and 323,282,836 people out of the current estimated world population of 8,082,070,900, have cognitive disabilities. Furthermore, it is important to consider that people with intellectual disabilities constitute a highly diverse and heterogeneous group, with each intellectual disorder presenting different manifestations. Generally, individuals with intellectual disabilities have difficulties with memory, problem solving, attention, and comprehension of others. Malachowski et al. [12], in their guide to the DSM-5 (Diagnostic and Statistical Manual of Mental Disorders) by the American Psychiatric Association (APA), aim to understand mental disorders. The guide identifies the specific needs of individuals with mental health conditions to address symptoms, related disorders, and risk factors. The study concludes with support recommendations to help these individuals and their families lead healthy lives that enable social integration. In summary, this guide is a key resource for understanding the general challenges faced by people with mental health issues. The data in Figure 1 show the need to create inclusive technological solutions.

Since more than 15% of the world’s population has some form of disability, according to data from the United Nations shown in Figure 1, it is estimated that approximately 12% of people with disabilities globally have some form of cognitive disability. This group faces significant barriers in accessing and using digital technologies, which limits their inclusion in society.

Technological solutions specifically designed for people with cognitive disabilities, although increasing in number, are still insufficient to meet the demand. While many applications focus on accessibility, only a small fraction is specifically targeted at people with cognitive disabilities, highlighting the urgent need to develop more inclusive and accessible solutions. In 2024, various organizations and platforms have begun implementing stricter accessibility standards, although progress toward full inclusion in digital spaces remains insufficient.

Kuo et al. [13] in their article, explore how people with disabilities perceive information and communication technologies (ICT). Despite advances in the development of accessible technologies, the study highlights the existence of significant gaps in digital accessibility. Many technological solutions are not designed with the diverse needs of people with disabilities in mind.

This study underscores the urgent need to improve and expand the available technological solutions to ensure that they are truly inclusive and accessible to all people with disabilities.

In this context, SmartRoutes emerges as an innovative and low-cost technological solution designed to provide comprehensive route management for individuals with cognitive disabilities in both indoor and outdoor environments. SmartRoutes focuses on enhancing route learning and navigation, facilitating the completion of routine tasks in work and social contexts. Additionally, the application is designed to address exceptional situations, such as emergency evacuations, ensuring that users can act effectively and be guided appropriately to the exit route, avoiding paralysis or chaos in high-stress situations. By providing an accessible and effective tool, SmartRoutes contributes to improving the autonomy and safety of individuals with cognitive disabilities across various scenarios.

## 2. Related Work

This section presents solutions for localization and guidance in indoor environments, focusing on assisting and locating users with or without cognitive disabilities, as well as other types of users, in both emergency and everyday situations. A key aspect is that these tools facilitate the creation of a network of pathways within buildings, such as office complexes, hospitals, and schools, to help people navigate and orient themselves indoors.

The following subsections primarily detail the proposed SmartRoutes Local Positioning System (LPS) solution. First, the technical concepts related to signal capture and the calculations necessary for real-time user localization using low-cost devices are addressed. Second, user interface considerations are discussed, focusing on creating an inclusive and accessible tool that can be used by both individuals with cognitive disabilities and those without. Finally, the specific technical aspects employed in the localization solution that motivated the creation of this paper are examined.

Regarding the type of user, SmartRoutes has been primarily designed for individuals with cognitive disabilities, such as Down syndrome, as this is the cognitive syndrome with the highest number of affected individuals worldwide (1 in 1000 to 1100 live births). According to the United Nations, between 3000 and 5000 children are born with this chromosomal disorder each year [14]. The solution has also been adapted for use by individuals with other types of cognitive syndromes, which are less common globally but are characterized by mild to moderate levels of disability, such as Prader–Willi, Williams, or Fragile X syndromes. These syndromes often cause problems with short- and long-term memory, mobility, comprehension, and others. Additionally, we have focused on behavioral characteristics and information interpretation that may affect these individuals’ stress levels in situations outside their comfort zones in order to design and develop a useful solution tailored to their cognitive needs.

In their article, Krasniqi et al. [15] conducted an extensive study on the current situation of people with cognitive disabilities, focusing on individuals with Down syndrome, as in our case. Krasniqi and their collaborators discuss how the life expectancy of this group has increased in recent years, as shown in Table 1, which presents the health challenges associated with aging in this population. These intellectual, psychological, and physical challenges, including visual and auditory deterioration, underscore the growing need for supportive tools for learning and navigation. Tools must be adapted to each person’s cognitive abilities, considering the varied life stages and associated challenges. Furthermore, the study shows that people with Down syndrome are enthusiastic adopters of technology, particularly mobile technology, which increases the potential for assistive tools. Table 1 presents the most commonly used assistive technologies for individuals with Down syndrome, including Augmentative and Alternative Communication (AAC) systems, augmented reality, navigation systems using beacons, and educational video games incorporating gamification elements.

**Figure 2:**
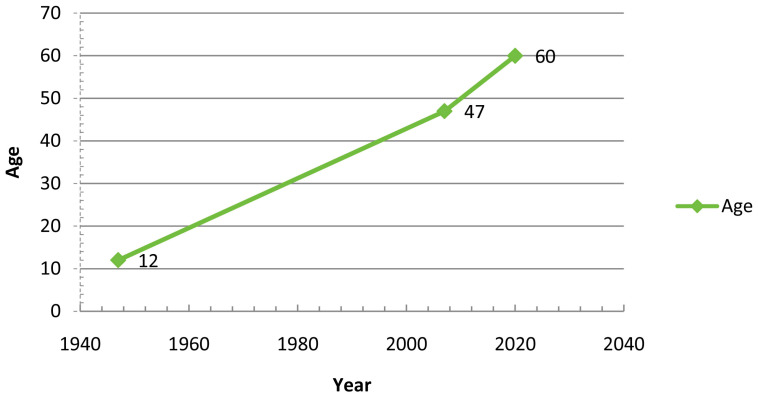
Graph of life expectancy for people with Down syndrome.

Figure 2 illustrates the significant increase in life expectancy for individuals with Down syndrome, rising by nearly 13 years over a period of 13 years. One of the main uses of indoor solutions is guiding users within large buildings that have numerous physical obstacles and a high volume of people, such as public buildings frequently used by various individuals with or without mobility challenges. Research in this area has led to solutions like the application developed by [16], utilizing LiDAR sensors, (sensors were invented by Hughes Aircraft Company in Glendale, CA, USA,) to collect data on measurements and angles. These sensors, controlled by robots, also capture RGB color data to create two-dimensional maps of indoor environments. This provides a comprehensive guide for users with mobility impairments, allowing them to navigate buildings not initially designed with accessibility in mind. The system creates a color video model of the mapped area, and with the use of Azure Kinect, (Microsoft Corporation, Redmond, WA, USA), it configures a detailed representation of the environment. This enables the production of two-dimensional maps of specific areas, laying the groundwork for a solution to guide users with mobility impairments through these spaces, effectively addressing architectural barriers.

In their article, Chen et al. [17] propose a structured analytical framework to synthesize the existing literature on pedestrian evacuation simulation (PES) approaches, models, and tools in indoor emergency situations. The objective of this framework is to provide a deeper understanding of the behavioral mechanisms during pedestrian evacuations and the necessary behavioral intervention measures to improve evacuation efficiency. Additionally, the study highlights the importance of developing high-fidelity and adaptable simulation tools that allow for more accurate evaluation of evacuation scenarios, thus contributing to enhanced safety during indoor emergencies.

Valizadeh et al. [18] present an indoor emergency evacuation system that integrates augmented reality (AR) technology to enhance guidance and assistance during critical situations. The proposed system addresses the limitations of satellite navigation systems, such as GPS, which do not function effectively indoors due to signal loss. Instead of relying on predefined maps, which are often limited to specific areas, this solution leverages Building Information Models (BIM), enabling its application to any structure with an available BIM model. For user localization and navigation within the building, a combination of Wi-Fi fingerprinting and the Pedestrian Dead Reckoning (PDR) method is used, utilizing smartphone sensors to calculate the user’s steps and direction with greater accuracy than other approaches.

Additionally, Nicolas A. et al. [19] conducted user tests with various commercial devices for locating and guiding visually impaired users within complex building structures. The study compared the performance of real-time narrative descriptions with memory-based conditions. Another solution was proposed by Pilski et al. [20], involving a system based on Bluetooth beacons and mobile devices to guide and assist people with hearing disabilities.

Finally, Dees and Dirks et al. [21] presented a small study involving four individuals with cognitive disabilities in a German city hall. The paper highlights the potential of smartphone-based indoor navigation apps to improve the independence and quality of life of individuals with cognitive or learning disabilities. The usability trial provided valuable insights into the effectiveness and user-friendliness of these apps in practical, real-world environments. Additional research focused on individuals with cognitive and visual disabilities was conducted by Lancioni et al. [22]. They carried out a study on an indoor guidance application with nine individuals with severe intellectual disabilities and blindness to assist them in traveling between different rooms. While Lancioni’s project relies on light sensor technology, our SmartRoutes solution incorporates features for both indoor navigation and emergency evacuation guidance. Our system not only assists individuals with cognitive disabilities in navigating within buildings but also supports evacuation procedures during emergencies. Furthermore, it offers guidance to users with mild to moderate cognitive impairments as they perform work-related or daily tasks, allowing them to navigate between rooms to complete specific cognitive tasks. In our previous research [23] in 2020, there were no studies focused on providing solutions to help locate and guide people in indoor environments during emergencies using mobile technology, especially for individuals with cognitive disabilities. However, recently, there has been growing interest in this promising field. We now find studies exploring and seeking technological solutions in this area. For instance, Zhou et al. [24] propose a solution to assist individuals in emergency evacuation by reducing the amount of information they need to process during search and hazard identification tasks. Another example is the article by Yoo et al. [25], which develops an emergency evacuation solution based on augmented reality. However, none of these studies consider cognitive accessibility.

## 3. Results of the SmartRoutes Architecture

Some studies emphasize indoor navigation for individuals with cognitive disabilities, particularly through mobile technology, to promote independence and improve quality of life. Our work distinguishes itself by offering a sensory navigation system that functions in both indoor and outdoor environments, providing route generation and detailed instructions. This comprehensive approach significantly enhances accessibility for individuals with cognitive disabilities in daily tasks and emergency situations. Our emphasis on sustainable technological solutions and inclusivity makes SmartRoutes a unique system designed to support the autonomy and well-being of individuals with cognitive challenges.

This paper presents several key contributions to the field of assistive technology for indoor navigation, particularly for individuals with cognitive disabilities:

- Innovative localization techniques: The proposed SmartRoutes Local Positioning System (LPS) introduces novel localization methods, enabling users to navigate complex environments with greater ease. Unlike existing systems, SmartRoutes leverages low-cost devices, making it accessible for widespread use in settings such as schools and hospitals.
- Inclusive and accessible design: The system’s user interface is designed to prioritize inclusivity and accessibility, ensuring that both individuals with cognitive disabilities and others can easily engage with the system. This focus on the user experience not only aids in everyday tasks but also significantly improves emergency navigation by providing critical guidance to safe exits.
- Addressing vulnerable populations: Our research focuses on the specific needs of vulnerable populations, such as individuals with Down syndrome, highlighting the social impact of assistive technologies. By incorporating insights from previous studies, we emphasize the urgent need for tailored solutions that can improve the quality of life for these individuals.
- Methodological approaches: We outline the methodologies used in developing SmartRoutes, emphasizing real-time localization techniques and adaptive features tailored to the cognitive challenges of users. Through user feedback and preliminary evaluations, we demonstrate the system’s effectiveness in reducing stress and improving navigational skills in both routine and emergency situations.

### 3.1. SmartRoutes Architecture

All the analyzed solutions share a common goal: to address current challenges in guiding and locating people in indoor environments, whether they have disabilities or not, and whether they are in emergency situations or everyday circumstances. Aligned with this objective, the SmartRoutes solution has been developed. SmartRoutes is an Android mobile application focused on the localization and positioning of users in indoor environments, with a focus on cognitive accessibility. The guidance applies to both everyday situations and emergency evacuations.

The application consists of three modules:

1. Outdoor location: This section uses maps based on OpenStreetMaps (OSM) connected to a “SmartRoutes Manager or SmartRM” (version 1.0) web platform, which communicates with the SmartRoutes mobile app via an external server. SmartRM is designed to allow users to generate outdoor routes. In SmartRM, a location is specified, such as a starting address (for example, the user’s home). This information is transferred to SmartRoutes and the app helps the user reach their destination, such as their workplace, using the OSM map.
2. Indoor location with emergency guidance: In the event of indoor emergencies, the SmartRoutes mobile app provides audio directions and on-screen information about the user’s location. It also alerts users to potential obstacles in the environment. The entire process of data storage and connection is performed autonomously on the mobile device, without the need to connect to an external server database. This ensures that in hostile emergency situations where communication may be disrupted, the SmartRoutes app continues to function uninterruptedly. The SmartRM platform also allows for the generation of routes and points for indoor locations.
3. Indoor activities: This section allows users to engage in cognitive activities to improve concentration. Users move through classrooms within a building, and the app informs them of their current location and the activity they should perform. Each activity has a time limit of 10 min. Once the time is up, all activities conclude and the user receives feedback based on the activity performed. In this case, communication and data storage are also managed within the mobile device through an embedded database.

It is important to highlight that the module designed for activities involving gamification was developed to explore in greater depth how individuals with Down syndrome respond to tasks presented by a technological device. It examines how they manage and respond to long and abstract instructions compared to concise instructions that require a primary survival reaction. For example, in the emergency module, instructions are often brief and descriptive, as the messages describe elements that users visually perceive in their current environment. The type of response required from the user in these scenarios is more instinctive and primarily involve receiving an input and needing to perform an action (action–reaction effect). However, in the activities module, the user must listen, understand, and analyze, formulating a strategy within a short period to carry out the proposed activities. In this case, the reaction is not as immediate; the user can take some time to mentally analyze what needs to be done. Additionally, each activity assesses the user’s level of concentration on the task and their comprehension of the messages informing them of the tasks to be completed.

In this regard, we refer to the article by Özbeşer et al. [26], where the authors analyze the effectiveness of two approaches: Cognitive Orientation to Daily Occupational Performance (CO-OP) and Conductive Education (CE) on the motor skills of a group of 12 children with Down syndrome. To do so, they engaged the children in activities related to school/productivity, self-care, and play/leisure, examining which of the two approaches yielded better performance when the children were given specific tasks requiring concentration.

In recent developments in indoor navigation systems, there is a growing interest in creating solutions that cater to the specific needs of individuals with cognitive disabilities. These advancements aim to enhance accessibility and independence for this demographic in various indoor environments. By leveraging technologies such as Bluetooth Low Energy (BLE) and mobile devices, researchers are exploring innovative approaches to provide personalized guidance and support for individuals with cognitive disabilities in both routine and emergency situations. Additionally, the integration of user-friendly interfaces and inclusive design principles is becoming increasingly important to ensure the usability and effectiveness of these indoor navigation systems.

**Figure 3:**
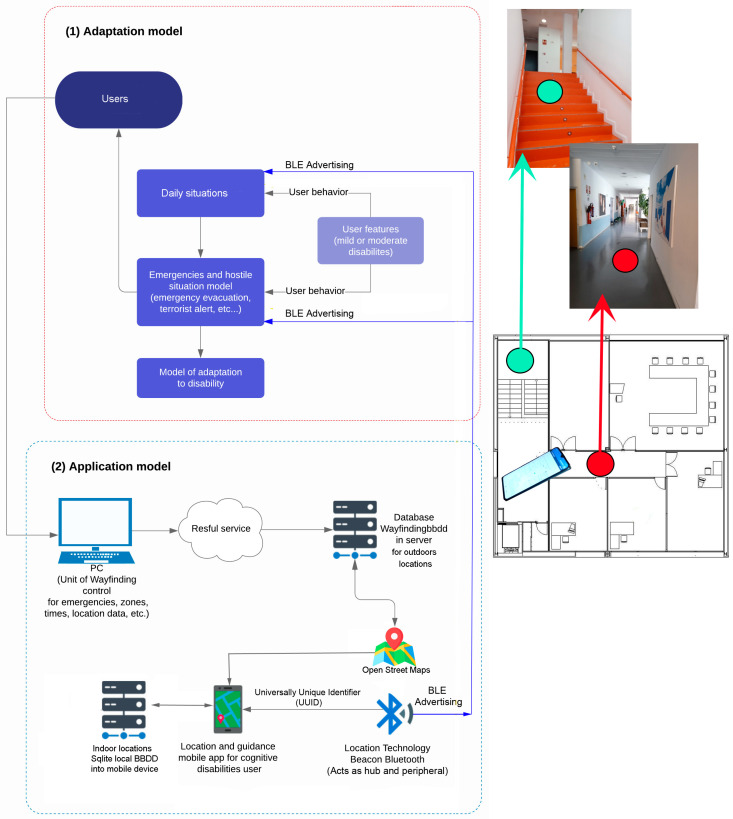
Architecture of the system proposed.

Figure 3 defines the final architecture of the guidance and localization solution based on Bluetooth Low Energy (BLE) technology. It represents the adaptation of users with mild to moderate cognitive disabilities to the solution and its usage environment under various environmental conditions, both in everyday situations and stressful circumstances involving hostile or emergency conditions.

In this model, two primary components are delineated. The outdoor component of the system is configured with high capacity, capable of accommodating up to 250 concurrent users while hosting both the database and web application. This configuration also supports the indoor environment, where users are not required to connect to a server for guidance due to the presence of a local SQLite database embedded within their devices. This feature ensures that the application remains autonomous and fully operational in emergency situations where communication disruptions may occur. The bidirectional location data obtained from Bluetooth beacons, which are essential for localization and guidance, are stored locally and synchronized with the server whenever feasible. For outdoor environments, a web platform is provided where users can input location requests, subsequently displayed on an OpenStreetMap interface via mobile devices. This configuration relies on the web server to efficiently manage and process location requests, ensuring accurate and timely display on the map interface.

The security and orientation information broadcast by the beacons, combined with user device position tracking data during movement, are stored in the server’s local database via the mobile device. This ensures that critical information required for both indoor guidance and outdoor localization is securely managed and accessible even during adverse conditions.

To adapt instructions to the specific needs of individuals with cognitive disabilities, the application focuses on providing simple and clear messages through both text and voice. These instructions are presented in short sentences and simple language, with an emphasis on intonation to aid memorization and the creation of mental maps. In hostile or emergency environments, where visual communication may be limited, audio messages are prioritized and complemented, when possible, by simple visual elements like graphics and images, in addition to text. This strategy of using simplified vocalizations and concise textual content seeks to ensure that instructions are both comprehensible and effective for individuals with cognitive disabilities, thereby enhancing their capacity for autonomous and safe navigation across diverse environments.

This study has addressed the urgent need to develop technological solutions that facilitate navigation and the management of daily activities for individuals with cognitive disabilities, particularly those with Down syndrome. Through the implementation of the SmartRoutes application, it has been demonstrated that it is possible to create personalized routes and platforms that not only optimize the execution of daily tasks but also promote effective learning in workplace environments.

In the context of the state of the art, there is a significant lack of accessible tools that integrate technology into the daily lives of individuals with disabilities. While there are applications and devices that address localization and guidance in indoor environments, few have been specifically designed to meet the needs of users with cognitive disabilities. This work stands out for its focus on accessibility and usability, employing a user-centered design that considers the capabilities and limitations of this population. The methodology adopted, which includes user testing in real environments, has validated the effectiveness of the application in both everyday and emergency situations, ensuring that the proposed solutions are practical and effective.

The differentiating elements of SmartRoutes include its ability to customize routes according to the individual needs of users, as well as its integration of Bluetooth BLE technology for precise localization. This technology not only enhances the user experience by providing real-time information but also allows users to interact with their environment more autonomously. Furthermore, the application has been designed to be intuitive, using clear language and voice messages that facilitate understanding, which is crucial for users with cognitive diversity.

Importantly, the results obtained from the testing phase, including detailed calculations of beacon localization accuracy and optimal scanning times, reinforce the effectiveness of the SmartRoutes system. These results demonstrate that the application can reliably locate users and provide timely guidance, which is essential for enhancing user confidence and independence in navigating both familiar and unfamiliar environments. The successful deployment of the system in various settings, as evidenced by the positive feedback from participants, further underscores its potential as a valuable tool for individuals with cognitive disabilities.

The implementation of SmartRoutes represents a powerful tool for managing routine activities, contributing to the autonomy of individuals with disabilities. By facilitating the execution of daily tasks and learning in workplace settings, this solution not only improves the quality of life for users but also promotes their social and labor integration. By providing an accessible means to navigate complex spaces, anxiety and stress associated with unfamiliar situations are reduced, thereby encouraging greater community participation.

In conclusion, this work not only offers an innovative solution to a persistent problem but also lays the groundwork for future research in the field of assistive technology. Future studies are suggested to focus on evaluating the application in a variety of contexts and with different user groups, as well as exploring new technologies that could further complement and enhance the navigation and activity management experience for individuals with cognitive disabilities.

#### 3.1.1. Development and Design of SmartRoutes

The SmartRoutes application, as mentioned earlier, has been developed on the Android platform using the object-oriented programming language Java. The foundational API version utilized for the application is Android API 33, also known as Android Tiramisu (API level 13).

**Figure 4:**
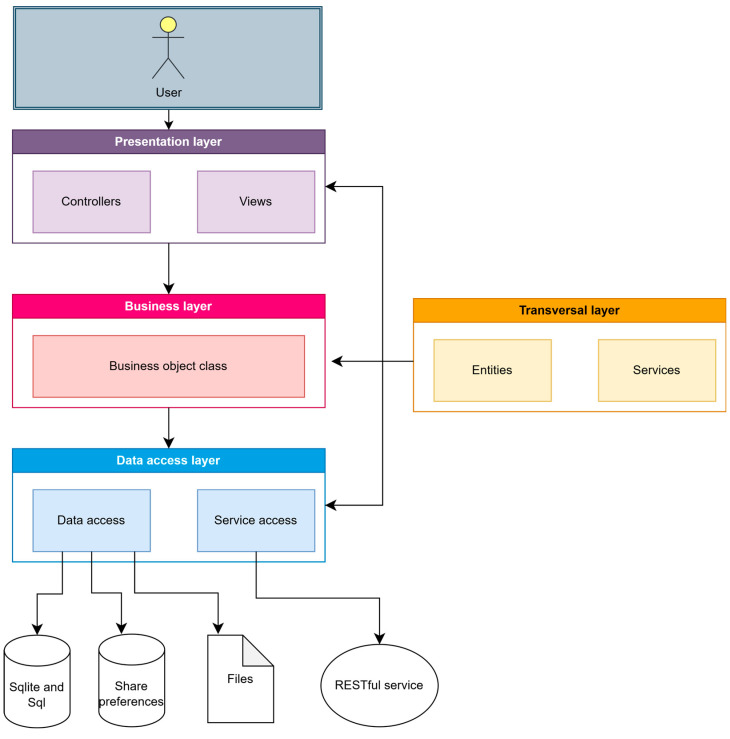
Layered architecture.

The SmartRoutes application is built upon the Android Multilayered Architecture model, defined by four interconnected layers: the application layer, framework layer, libraries, and kernel. Following the general multilayer architecture of Android, this application employs Java packages that structure the project into multiple logical units or layers. This approach aids in separating different project responsibilities. Figure 4 presents a diagram illustrating the project responsibilities or layered architecture.

Each layer has a function that allows structuring the project:

- Presentation layer: This layer is responsible for presenting information to the user. Data processing to display to the user is performed in this layer, as well as the presentation of views that constitute the application’s lifecycle flow.
- Business objects layer: This layer connects the presentation layer with the data access layer, validating that the data passed as parameters comply with the rules established by the application.
- Data access layer: This layer is responsible for data manipulation, in-memory storage, and retrieval and updating of information.
- Cross-cutting layer: In this layer, objects and services used throughout the other layers are defined. For example, classes for a music playback service are defined within a content class in a cross-cutting package.

#### 3.1.2. Localization and Guidance System 

The SmartRoutes guidance solution utilizes beacons that communicate via iBeacon and Eddystone protocols through Bluetooth for user localization. These beacons have an approximate range of 10 m, but in large spaces, multiple beacons are required to ensure signal coverage across the area. It is important to note that the architecture will be evaluated in a space designed to assess localization and guidance for training daily tasks, such as work-related activities: going to the office, delivering documents, visiting the restroom, clocking in, etc., in a sequence determined by the entity aiming to assist in the user’s training. An example of deployment or evaluation is illustrated in Figure 3, where the spaces can be further elaborated upon.

**Figure 5:**
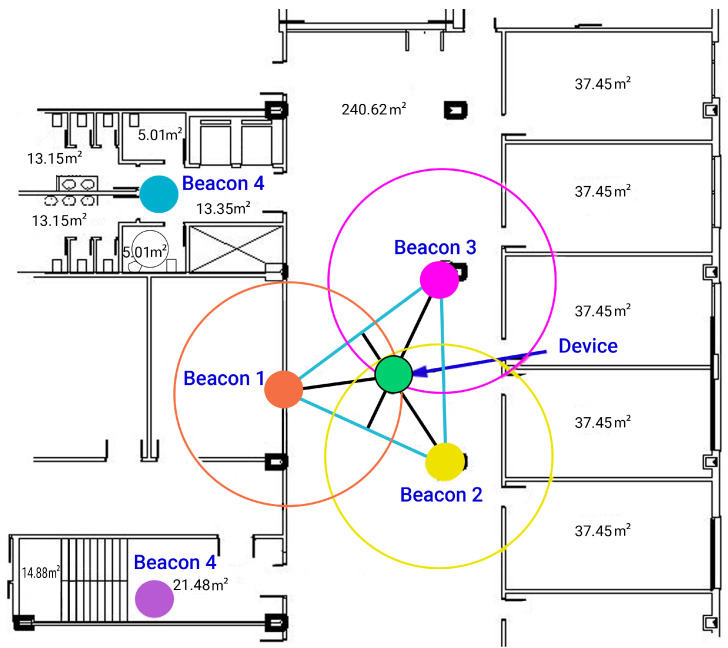
Positioning of beacons in a specific building at University Rey Juan Carlos.

Figure 5 depicts the placement of beacons in a specific building during a test conducted with the application. The filled circles represent the effective coverage area of each beacon, indicating the maximum range within which a device can reliably detect the beacon signal. The hollow circles depict the limit of the theoretical range of beacon detection, showing areas where signal strength may start to diminish but could still provide partial coverage. In the vestibule area, measuring 240.62 m^2^, it was determined to position three beacons in a configuration where they are closely spaced within the 10m signal emission range. A triangle was formed based on these positions to facilitate trilateration. The centroid or barycenter of the triangle, where the three medians intersect, signifies the point where the mobile device receives the strongest RSSI intensities. In confined spaces, a single beacon is typically adequate.

This method operates with x and y coordinates, necessitating the initial measurement of distances from the mobile device to each beacon used in the trilateration method.

#### 3.1.3. Trilateration and Intensity Calibration

To perform localization using Bluetooth, as mentioned earlier, the trilateration method has been employed. This method works with coordinates x and y, and to obtain these coordinates, it is necessary to know the distances from the mobile device to the beacon.

#### 3.1.4. Calculating the Distance Between the Beacon and the Mobile Device

The calculation of the distance between both elements, beacon and mobile device, is carried out by obtaining the RSSI indicator from the beacon. Once this value is obtained, a reference value, denoted as *r*, and three fixed coefficients, denoted as *a*, *b*, and *c*, are used. These coefficients are the parameters used to simplify the system of equations and find the coordinates (*x*, *y*) of the unknown point. Therefore, the equation will be Equation (1):

(1) $$d=a\times \frac{RSSI}{r}b+c$$

Once the beacons and the smartphone are positioned in the designated areas, measurements of RSSI intensities from the smartphone to the beacons are taken at various distances ranging from 0.25 m to 12 m. This process is facilitated using a laser distance meter or tape measure, along with a mobile application or RSSI intensity reader device. The TX Power configuration of the beacon has been set to 7 (4 dBm).

Regarding the calculation of the distance between both elements, it is carried out by obtaining, as mentioned earlier, the RSSI indicator from the beacon. Table 2 shows the correlation between distance and intensities.

To conduct the measurements accurately, it is essential to establish a reference value, which corresponds to the Received Signal Strength Indicator (RSSI) at a distance of one meter. Due to variations inherent in the Bluetooth range of different devices, the RSSI value recorded at one meter will serve as the baseline reference for the smartphone used in the tests. Each smartphone model may produce a distinct reference value at this distance, influenced by factors such as the chipset and the efficiency of the Bluetooth antenna. In this instance, the established reference value is −59 dBm.

Once the RSSI value is obtained, the reference ratio *rt* can be calculated using Equation (2), represented as follows:

(2) $$rt=\frac{RSSI}{-59}$$

This equation represents the reference value divided by the previously obtained RSSI values at varying distances. Table 3 and Table 4 illustrate these calculations.

Next, the two fixed coefficients, *a* and *b*, are obtained; for this, a potential regression in nonlinear R will be carried out with the reference values and the RSSI data. We will assign the value (*rt*) and the value in meters as *x* and *y* coordinates. Table 4 shows the calculations of *x* and *y* values.

The power regression (PowR) for these data is Equation (3):

(3) $$y=1.0229x^{0.1731}$$

The coefficient values are a = 1.0229 and b = 0.1731. The values from the distance method between the beacon and the mobile device, implemented in the Java class responsible for distance calculations during the indoor beacon scanning operation in the SmartRoutes application, incorporate these coefficient values. Table 5 presents the predicted distance calculations for the coordinates a and b.

Subsequently, an approximate distance will be determined using the coefficients a and b, which will facilitate the calculation of the coefficient c, as delineated in Equation (4). In this context, d represents the estimated distance between the beacon and the mobile device. This value is derived from the Received Signal Strength Indicator (RSSI), which quantifies the strength of the signal received by the mobile device from the beacon.

(4) $$d=a\times {\left(\frac{RSSI}{rt}\right)}^{b}$$

The coefficient c is a zero-intercept variable.This variable optimizes the estimation at 1 m; therefore, the calculation is performed by subtracting the actual 1-m distance and the approximate 1 m distance, as shown in Equation (5):

Coefficient*c* = (1 − 1.031719637) = −0.031719637 (5)

To finish with the data of the three coefficients a, b and c, we obtain the approximate distances. Table 6 shows the predicted distance calculation of the c coordinate, as shown in Equations (6) and (7).

(6) $$d=a\times {\left(\frac{RSSI}{rt}\right)}^{b}+c$$

*c* = −0.031719637 (7)

#### 3.1.5. Trilateration Calculation

**Figure 6:**
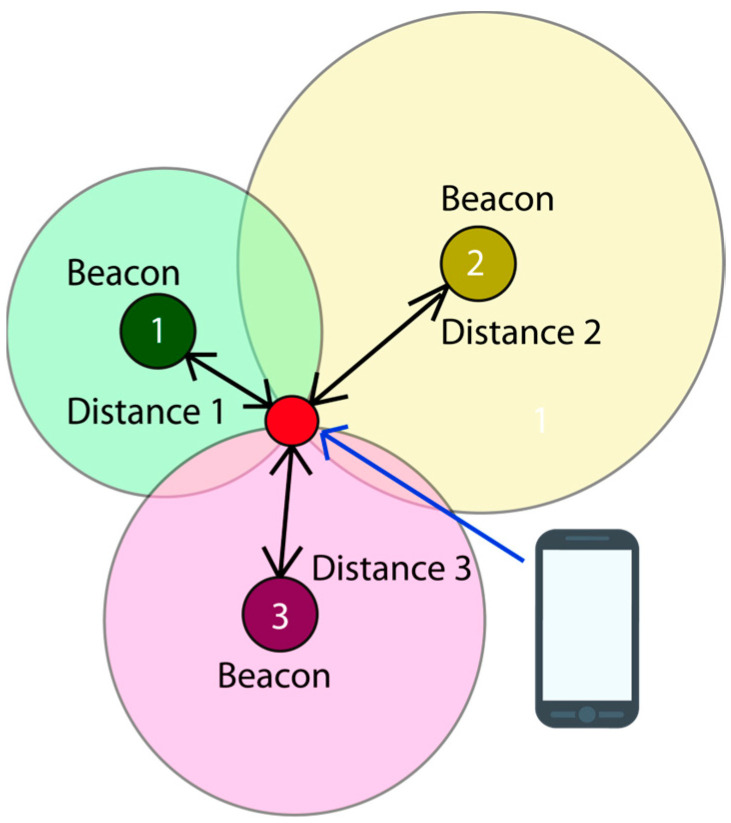
Trilateration beacons and device.

Trilateration is a geometric technique to determine the position of an object, in this case, a mobile device, by knowing its distance to three reference points, in this case, beacons. Figure 6 shows an example of trilateration with beacons and a mobile device.

The position is calculated by determining the difference in distances between waypoints 4 and 5. For ease of identification, we label the waypoints with letters, corresponding to waypoints (*b* and *c*) and (*b* and *a*), as shown in the following equation, Equation (8).

(8) $$pa={da}^{2}-{dc}^{2},\;pb={db}^{2}-{da}^{2}$$

Finally, the coordinates y and x are obtained, as demonstrated in the following equation, Equation (9):

(9) $$y=\frac{pb\left(xb-xc\right)-pa(xb-xa)}{\left(ya-yb\right)\left(xb-xc\right)-\left(yc-yb\right)\left(xb-xc\right)},\;x=\frac{y\left(ya-yb\right)-pb}{\left(xb-xc\right)}$$

**Figure 7:**
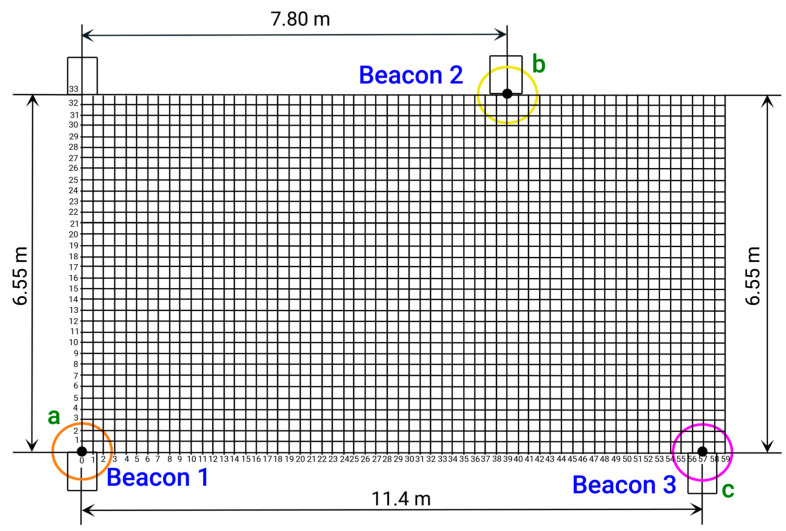
Cartesian coordinate plane trilateration beacons at laboratory III, University Rey Juan Carlos.

A Cartesian coordinate plane is utilized below to determine the position and location of beacons 1, 2, and 3 (or *a*, *b*, and *c*) in the trilateration area located in the lobby of the specified building. The vertices of the triangle formed by the trilateration correspond to the coordinates (*x*_1_, *y*_1_), (*x*_2_, *y*_2_), and (*x*_3_, *y*_3_) of the known points from which distances are measured. Figure 7 and Table 7 display the Cartesian plane and the coordinate data table, with the origin point (0, 0) located at the bottom left corner of the Figure 7.

## 4. Methodology for Evaluation

The SmartRoutes system is an advanced solution designed for location tracking and navigation across different environments. It encompasses three key modules: exterior location tracking, interior location tracking for hostile and emergency situations, and indoor activity management. This chapter details the methodology employed for evaluating the SmartRoutes system, which includes the development and testing phases. A flowchart illustrating the evaluation process is provided to offer a clear view of the methodology.

### 4.1. System Overview

#### 4.1.1. Functional Modules

(a) Outdoor location module: This module provides navigation capabilities for outdoor environments using global positioning technologies, such as GPS (Global Positioning System) or GNSS (Global Navigation Satellite System). It is responsible for determining the user’s precise location, calculating optimal routes, and providing real-time turn-by-turn guidance. The module ensures that users can navigate complex outdoor environments, such as city streets, parks, and public transportation systems, with ease. It can also include features like location sharing, geofencing, and integration with public transportation schedules to offer more robust navigation and mobility solutions.
(b) Indoor location module for hostile and emergency situations: This module is specifically designed for navigation in critical indoor environments, such as hospitals, factories, or buildings experiencing emergency situations (e.g., fires, earthquakes). It utilizes a combination of technologies like Bluetooth Low Energy (BLE) beacons, Wi-Fi triangulation, and inertial sensors to offer accurate real-time positioning where GPS signals are unavailable. This module is crucial in ensuring that individuals, especially those with cognitive disabilities, can navigate safely and efficiently during emergencies. It may also incorporate features such as emergency exit routes, real-time danger notifications, and guidance to safe zones within the building.
(c) Indoor location module for cognitive activities: This module is designed to assist users with cognitive disabilities in performing routine tasks within indoor environments, such as schools, offices, or shopping centers. It not only provides optimized routes but also offers personalized recommendations and reminders to help users complete specific tasks. The module is tailored to simplify navigation for individuals with cognitive challenges by breaking down complex tasks into manageable steps and offering cues (visual, auditory, or haptic) based on the user’s preferences and needs. It ensures that users can independently navigate both familiar and unfamiliar environments, thereby improving their autonomy in daily life.

#### 4.1.2. Web Management Platform—SmartRM

SmartRM serves as the backbone of the entire system, providing a user-friendly web interface for managing and creating routes in both outdoor and indoor environments. The platform allows caregivers, administrators, and other responsible parties to input route data, configure specific guidance parameters, and monitor users’ progress in real time. It supports features such as creating customized routes for individual users, setting task reminders, adjusting navigation preferences based on cognitive ability, and storing a history of completed tasks and routes for review. 

SmartRM collects and processes information from various sources, such as outdoor and indoor location modules, centralizing it for efficient data management. It also includes capabilities for handling user profiles, including their preferences, cognitive levels, and required support. Additionally, it enables administrators to monitor system performance, ensuring accurate and up-to-date information is delivered to users through the mobile application. The platform is scalable, allowing integration with multiple buildings or environments, making it suitable for use in smart city projects, large facilities, and organizations supporting people with disabilities.

#### 4.1.3. Native Mobile Application—SmartRoutes

The mobile application, developed using Android programming, serves as the user interface for individuals navigating both indoor and outdoor environments. It receives data from SmartRM and translates it into actionable instructions tailored to the user’s cognitive abilities. The app is designed with a simple and intuitive interface, using large icons, clear text, and voice commands to ensure ease of use for individuals with mild to moderate cognitive disabilities. It offers step-by-step navigation instructions and adapts to the user’s context, providing additional support as needed, such as reminders for task completion or notifications about nearby landmarks.

SmartRoutes also includes personalization features, allowing users to adjust the level of guidance, such as enabling or disabling certain types of notifications (e.g., auditory or haptic feedback) or adjusting the complexity of instructions. The app integrates with the phone’s accessibility settings to further enhance usability, supporting text-to-speech, voice control, and screen readers. In emergency situations, SmartRoutes can switch to providing critical safety information, helping users quickly locate exits or safe zones. Additionally, the app includes real-time updates for changing conditions, such as delays in public transport or emergency alerts within indoor spaces.

### 4.2. Evaluation Methodology

#### 4.2.1. Development Phases

The evaluation of the SmartRoutes system followed a structured approach, including the following:

(1) Planning: In this phase, an evaluation of the state of the art of localization and positioning technologies currently available was conducted. We also analyzed solutions that support hostile emergency situations in general and those intended for people with Down syndrome or syndromes like Prader–Willi, Williams, or Fragile X syndromes, of mild to moderate severity. As a result of this exhaustive research, the aforementioned article [23] was created. In this article, we highlight the lack of solutions aimed at providing support in emergency situations and emphasize the need to create solutions in this regard, broadly defining the purpose of our future solution and the objectives we would pursue to achieve it.
(2) Requirement definition or analysis: In this second stage, we defined the functional and non-functional requirements of the solution, which were also outlined during the planning phase in the aforementioned article [23]. We identified the basic accessibility and usability requirements, as well as functions that, in our judgment and according to the research we conducted regarding the cognitive needs of our users, should be implemented in our solution, such as colors, icons, and textual elements that are visually legible and audible, considering the physical difficulties these individuals with cognitive disabilities present.
(3) Design and prototype: In this phase, we defined the architecture design that we needed to model to carry out our solution. We determined some design aspects, such as the user interface, and decided to use a web platform, SmartRoutes Manager or SmartRM, from which we would manage points of interest. We defined two main modules: the outdoor localization module and the indoor localization module. We also decided to use a mobile application that would run on the Android SmartRoutes programming language, which would communicate with the SmartRM web platform. Additionally, we determined that the indoor localization section would include a third cognitive activities module, where users would be prompted to perform a series of activities to evaluate their level of concentration and comprehension. We decided to use Bluetooth beacons as a peripheral element to help locate and position the user at all times within buildings.
(4) Software development: In this stage, the software itself was created based on everything previously defined. Some changes were made, such as the creation of a local database within the mobile application, so that in situations where communication with the server is lost or there are communication problems with the internet in hostile environments, the integrity of the application would not be compromised, allowing it to continue providing support to users. The appropriate algorithms and data structures to be used were also defined.
(5) Testing: In this stage, the software was tested to identify and quickly fix errors, ensuring that the entire system functions properly and meets the users’ needs.
(6) Release: Once the project has passed all evaluations, it is made available to end users.
(7) Maintenance: This is possibly the most important stage since the use of the software allows for the detection and elimination of defects, as well as the adaptation or addition of new requirements and functionalities to the solution.

#### 4.2.2. Testing Process

The evaluation process encompassed the following:

(1) Simulated environment testing: assessing the system in controlled conditions to detect and rectify issues before real-world implementation.
(2) Real-world environment testing: implementing the system in actual settings to validate its performance and functionality in practical scenarios.
(3) Usability evaluation: reviewing the interface and user experience to ensure accessibility and satisfaction for users with cognitive disabilities.

In the simulated environment testing, SmartRoutes was compared to existing indoor localization and guidance systems, including traditional Bluetooth-based trilateration methods and Wi-Fi-based positioning systems. Key performance indicators, such as localization accuracy, response time, and user satisfaction, were evaluated.

SmartRoutes showed superior performance in terms of real-time responsiveness, with beacon signals being processed in less than 3 s on average, compared to 5–7 s in standard trilateration methods. Additionally, the system’s ability to guide users to safe exits during emergency situations was found to be 20% more efficient than conventional Wi-Fi-based systems, as indicated by shorter times for reaching evacuation points in simulated fire drills.

Moreover, the integration of cognitive accessibility features in SmartRoutes, such as audio and visual guidance tailored to users with cognitive disabilities, provides a significant improvement in user satisfaction and usability. This stands in contrast to existing systems, which often lack features specifically designed for individuals with cognitive impairments, leading to higher error rates and user frustration in emergency scenarios.

### 4.3. Flowchart of the Evaluation Process

**Figure 8:**
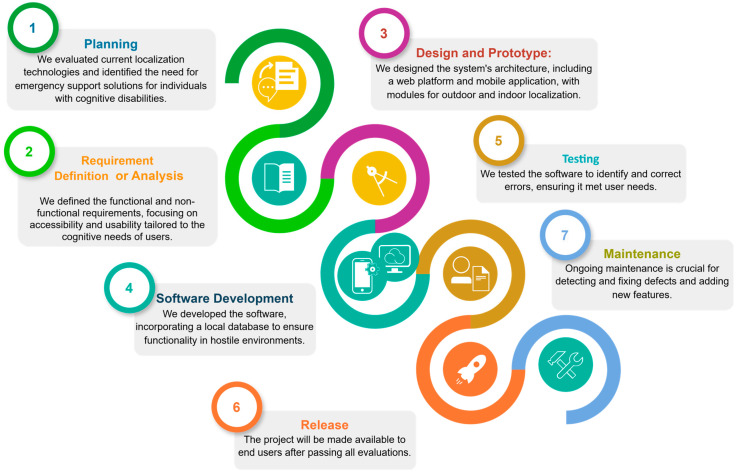
Project phase diagram for testing.

Figure 8 shows the project phase diagram, which provides a visual representation of the evaluation methodology for the SmartRoutes system. It outlines each stage of the process, from requirement analysis to final evaluation, highlighting the iterative nature of the development and testing phases.

#### 4.3.1. Testing of SmartRoutes:Use Cases

Two successful user tests for localization and guidance have been conducted in a residential building and in a specific building at University Rey Juan Carlos. For these tests, the beacons were configured.

The beacon transmission power (TX power) has been configured, as the higher the transmission power, the greater the beacon’s range. To configure the RSSI (Received Signal Strength Indicator) and avoid signal overlaps between beacons, the beacons are set to a value of 7 (4 dBm), which corresponds to −59 RSSI. After testing, we confirmed that this configuration is adequate, considering the conditions of the environments where the beacons are located and the materials that may attenuate the signals. It is unnecessary to increase the signal intensity by expanding the RSSI. The system operates with the Bluetooth BLE, iBeacon, and Eddystone beacon protocols. Given that the beacon system allows multiple protocols to be activated simultaneously and considering that the Eddystone protocol uses a calibration value of 0 m rather than 1 m like iBeacon, it has been deemed better to measure the beacon power at 1 m and then adjust the measurement by complementing it with −59 dBm.

The SmartRoutes application is configured to read the beacon scan every 3 s. Therefore, we need to set up our beacon system to emit signals every 300 ms, allowing us to obtain between 9 and 10 beacon readings. We acknowledge that the current update interval of 20 s (for emergency use case, given that beacons are detected between 14 and 17 s) and 30 s for the indoor activity use case may not match the rapid localization offered by some state-of-the-art systems. However, our system is specifically designed for users with cognitive disabilities, for whom simplicity and clarity are essential. The slower update rate ensures that instructions are clear and digestible, reducing the likelihood of confusion in stressful situations such as emergencies. Furthermore, our system remains operational even in the absence of network connectivity, ensuring that users receive reliable guidance in critical moments. Future improvements may include optimizing the update frequency to enhance responsiveness while maintaining ease of use for the target user group.

##### First Test in a Residential Building

**Figure 9:**
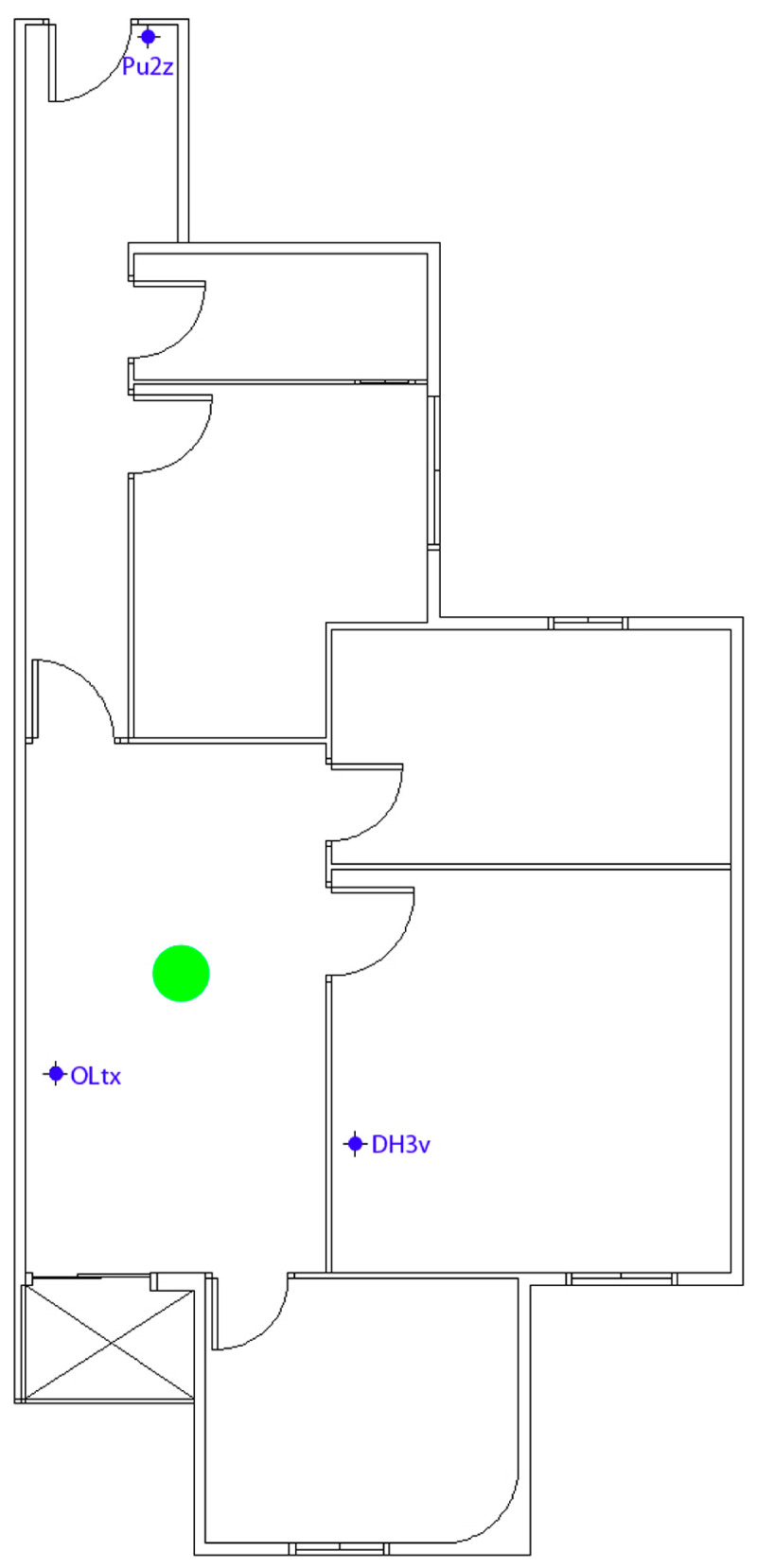
Tests in residential building locations with 3 beacons.

A preliminary test was successfully conducted in a building using three beacon devices. The test involved placing three beacons in three rooms of an apartment (beacons PU2z, OLtx, and DH3v)—in the hallway, the dining room, and one of the rooms designated as Room 1. With the device in hand, a user navigated through the apartment, and in each room, the device accurately detected the beacon’s location, thus pinpointing the user’s position. The device displayed a user location map on its screen, with the user represented by a green circle. This information was shown on the mobile device’s screen, along with three text fields: one indicating the user’s current location (hallway, dining room, or Room 1), another indicating the building floor, and a description of the room. All information was presented textually, and an audio prompt also informed the user about the room’s description. For example, in the hallway, the user heard a prompt indicating they were in the hallway on the first floor, along with information about important elements located around the user at that instance, such as doors and windows, as shown in Figure 9.

**Figure 10:**
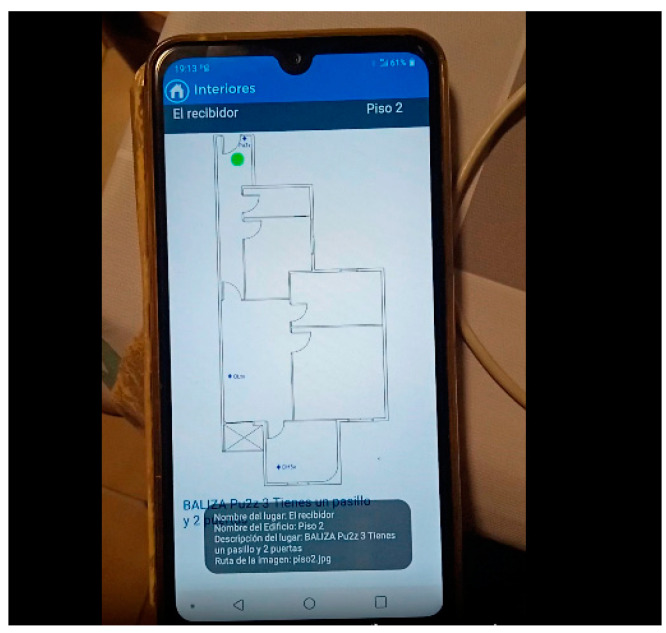
Testing in residential building locations.

During this test, we analyzed the application’s performance during navigation, including scan times and detection of room changes. Figure 9 and Figure 10 depict the test map and the layout of the beacons, along with images showing successful tests inside the apartment.

During testing in the residential building, as shown in Figure 9 and Figure 10, we analyzed the application’s performance in terms of navigation, scan times, and detection of room changes. Despite the overlapping coverage of beacon signals, the application consistently maintained accurate readings of the user’s location without affecting the clarity or reliability of the displayed information. The overlapping beacon signals, configured with an RSSI of –59 dBm, do not interfere with the scientific validity of the results, as the. y are calibrated to ensure precise localization in each room without causing signal confusion or data misinterpretation. Thus, the overlap has no negative impact on the application’s usability or the scientific interpretation of the results. Table 8 shows the times it takes for the application to display the user’s location in each room of the floor during the real-time floor test.

Table 9, in the use case of a sample, shows the messages or indications given to the user in case of emergencies according to the situation and structure of the interior of the building where the test is carried out, and Table 10 shows the instructions for the activities that the user must perform inside the building.

**Figure 11:**
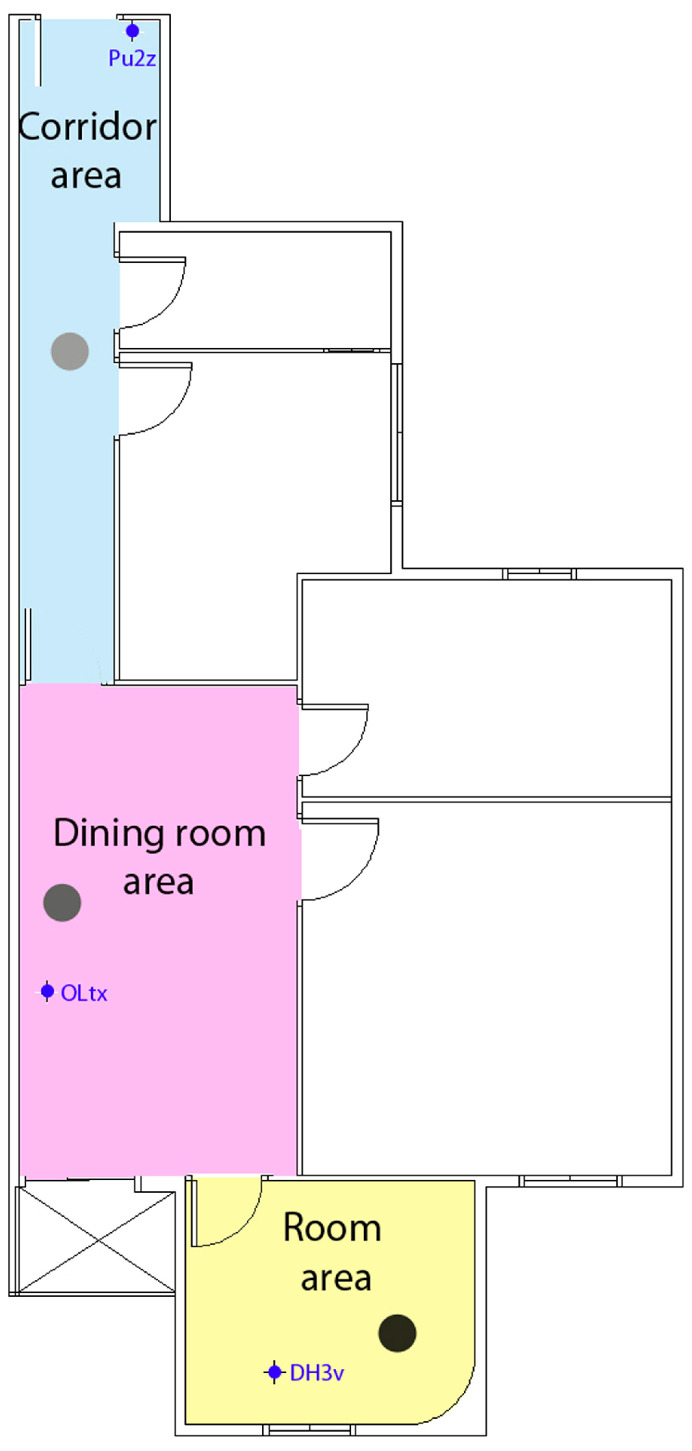
Areas of the residential building.

Figure 11 shows the areas of the residential building applicable in both use cases: emergency evacuation or functional activities.

##### Second Test at Rey Juan Carlos University

Second tests are conducted in a classroom at University Rey Juan Carlos. For these tests, five beacons were used and each will be identified according to the manufacturer-assigned identification name. They will be named “kontakt” to distinguish them in case there are other Bluetooth devices in the area and to facilitate filtering during debugging with Android Studio, along with a number according to the floor plan.. This area provides access to two stairwell and restroom areas where two beacons will be placed, one in each area, to verify accurate localization in those enclosed spaces outside the trilateration area.

Since the lobby area measures 240.62 m^2^, it has been decided to concentrate the beacons in a layout where they are approximately 10 m apart for signal emission. Triangulation is established by constructing a triangle at these positions. The centroid of this triangle, where the three medians intersect—meaning the segment that connects a vertex of the triangle with the midpoint of the opposite side—is identified as the point where, according to calculations, mobile devices would receive the best RSSI signal intensity. During testing, we verified that these assumptions were correct.

**Figure 12:**
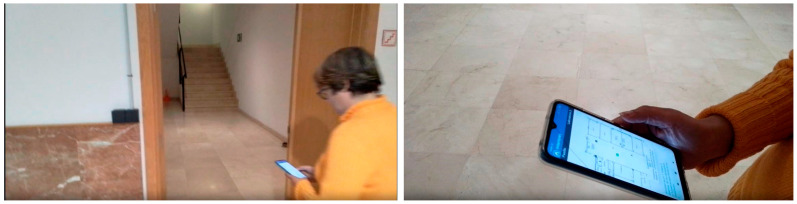
Plan for the location of the test beacons in the building at University Rey Juan Carlos.

**Figure 13:**
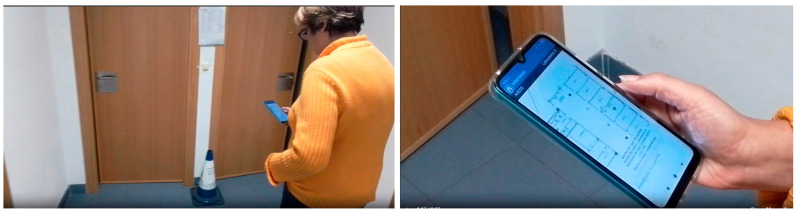
Layout of the location of the test beacons in laboratory III (Public Building) at University Rey Juan Carlos.

The user with the application in her smartphone or tablet conducted a route throughout the designated area of the building for the test. In this case, the behavior of the application in the environment of a basement with an extensive vestibule area covered by three beacons arranged in a trilateration form was analyzed, along with two more isolated beacons in two adjoining rooms. The study considered construction materials that could either dampen or not dampen the propagation of Bluetooth signals. Additionally, the accuracies of the beacons and the Bluetooth 5.0 version used in this scenario were analyzed, as well as the scanning times and results. Figure 12 and Figure 13 show the test layout and the placement of the beacons in the building of the university.

Several aspects have been examined during the initial user tests, including a preliminary assessment of how building materials impact signal dispersion. These initial findings have provided useful insights into how the application performs in specific environmental contexts. However, to better understand and optimize performance under a broader range of conditions, including different material compositions and building layouts, a more detailed and comprehensive analysis will be conducted in future tests.

1. The performance of the application was analyzed in a large space with multiple adjacent rooms and several beacons distributed across various compartments. This analysis included scenarios involving signal overlaps, detection accuracy, and the influence of building materials on signal strength. Additionally, the behavior of the application as users moved between floors was studied.
2. Further, aspects related to the operation of the guidance solution were evaluated, such as scanning times and the speed of data updates. Given that the application operates autonomously without server dependency, this evaluation has helped us identify how the system performs in more challenging scenarios.

In Table 11 and Table 12, the times taken by the application to display the user’s location in each room during real-time tests are shown.

Table 13 shows the messages or indications given to the user in case of emergencies according to the situation and structure of the building where the test is carried out. Table 10 shows the instructions for the activities that the user must perform inside the building.

**Figure 14:**
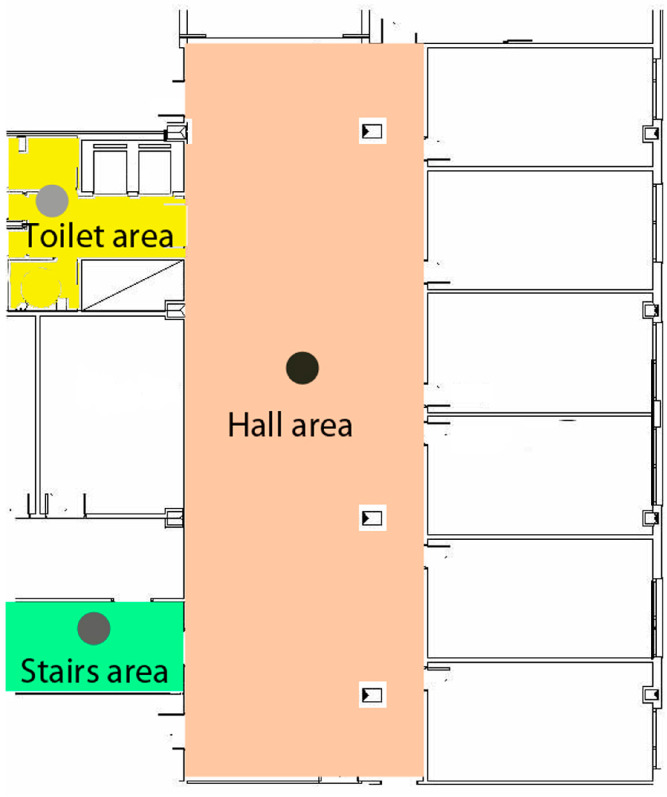
Areas of lab III for emergency proof.

**Figure 15:**
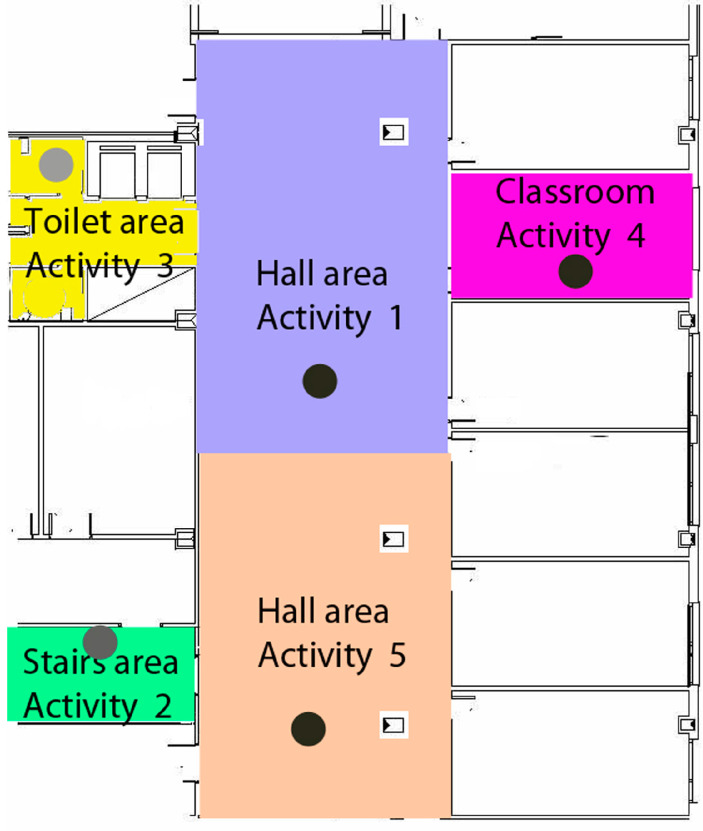
Areas of lab III for functional activities.

Following the layout shown in Figure 14, and Figure 15 displays the general distances where the beacons are placed in the vestibule area of Laboratory III, the most open area for beacon trilateration. This area provides access to two stairwell and restroom areas where two beacons will be placed, one in each area, to verify accurate localization in those enclosed spaces outside the trilateration area.

The proof shows the areas in the university building in case of emergency evacuation.

Figure 15 shows the areas in the university building in the use case of functional activities. Future user tests will analyze various aspects, including the following:

1. The communication between the user and the application will be analyzed in detail to determine if the application is understandable and accessible for users with mild to moderate cognitive functional diversity, and if it assists them in their daily activities. This analysis includes observing their behavior during the application’s usage test to assess the usability and comprehensibility of voice messages, text messages, layout, and interface design.
2. The application’s performance will be analyzed in a large space containing multiple adjacent rooms and a significant number of beacons distributed across different areas. Factors such as signal overlap, the application’s capacity to accurately detect signal strengths, and the impact of building materials on signal dispersion will be examined. Various environmental factors will also be considered, along with the application’s performance as the user moves between floors within the building.
3. Finally, scanning times, data update speed, and performance in scenarios involving multiple floors will also be analyzed.

Table 14 presents the instructions or indications provided to the user regarding the functional activities to be performed inside the building. These activities are designed to address different areas of cognitive development. Specifically, one activity promotes the stimulation of psychomotor skills, another stimulates attention and memory, a third activity encourages language development, and finally, an activity focused on spatial orientation improvement.

### 4.4. Conclusions from Testing at URJC and a Residential Building

Testing of the SmartRoutes system at Rey Juan Carlos University (URJC) and a residential building has demonstrated its effectiveness in navigation across diverse environments. In particular, the modules designed for indoor navigation for cognitive activities and for hostile and emergency situations performed exceptionally well. The iterative methodology employed during development, along with the thorough testing conducted, has ensured the system’s robustness and adaptability in various use scenarios. These tests validated the system’s ability to provide precise and useful navigation in real-world contexts, meeting established requirements and adapting to the specific needs of users in different environments.

## 5. User Interface

The SmartRoutes application features a shallow user navigation interface, meaning it does not require extensive navigation across multiple screens to reach the functional screens of the application. It uses buttons with graphics representing the category of each functionality. In the top menu bar of each screen, there are buttons that allow the user to close the application or return to the home screen.

**Figure 16:**
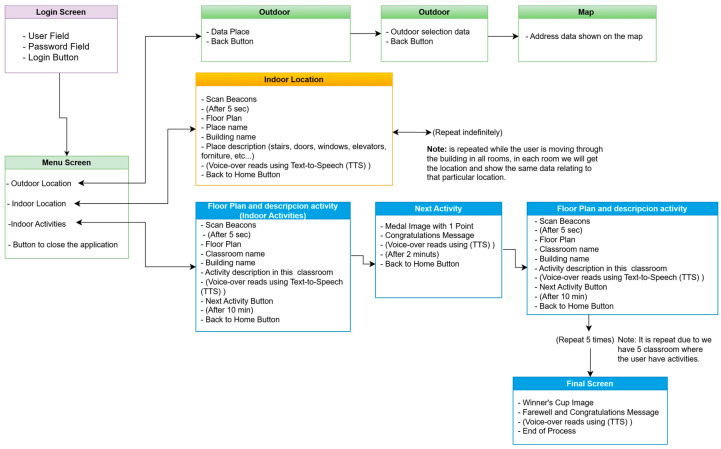
Blocks diagram of SmartRoutes application.

The user interface of this guidance solution is tailored to the specific needs of users who struggle with symbolization and abstraction. Therefore, the instructions provided by the application are simple, using accessible vocabulary, mainly through text and voice messages consisting of short sentences and plain language. Emphasis is placed on intonation, always following consistent patterns to aid memorization and the creation of mental maps. In hostile or emergency environments such as smoke, fire, etc., and during cognitive activities and outdoor guidance, audio messages are the most effective means of communication, supplemented by simple visual messages (graphics and images) to reinforce the message, if the environment allows. Figure 16 shows the block diagram between the different screens of the SmartRoutes application, including the options available on each screen and their functionality.

**Figure 17:**
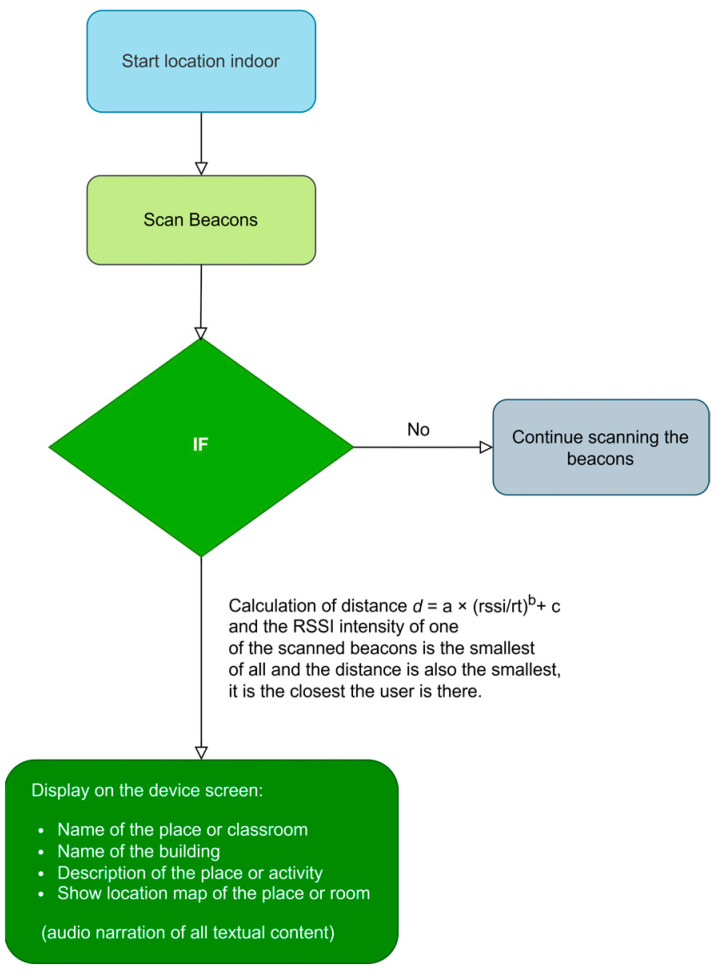
Flowchart, indoor location for emergencies and activities.

Figure 17 is the flowchart that illustrates the sequence of actions carried out by the SmartRoutes application to complete the task of displaying the user’s exact location within the building, whether for carrying out proposed activities or for guiding them during an emergency evacuation.

### 5.1. Graphic Design and Mobile User Interface

Below, the elements that make up the structure, UI, and functionalities of the mobile application are described. Graphically, self-explanatory icons have been used with soft colors predominantly in shades of blue, which in terms of color psychology convey calmness. This facilitates interpretation by our main users, who have mild to moderate cognitive functional diversity. For menu access or buttons, images and textual content are used, with text fonts Montserrat and Roboto, categorized as sans-serif typefaces, meaning they lack the small finishing strokes known as serifs. This choice aims to enhance readability.

#### 5.1.1. Icon of the Mobile Application

**Figure 18:**
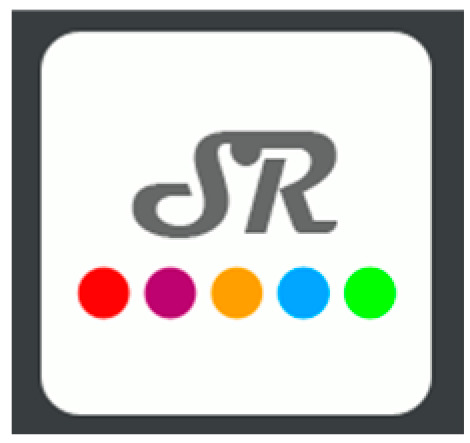
Rectangular SmartRoutes application icon.

**Figure 19:**
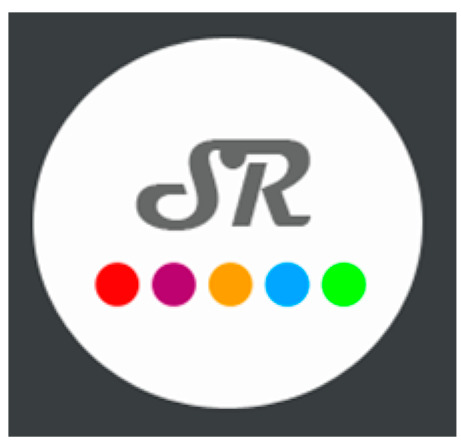
SmartRoutes circular application icon.

The application is represented by a graphical icon with a white background, featuring several colored circles and the application’s initials, “SR” (SmartRoutes), with the title displayed at the bottom of the icon. Figure 18 and Figure 19 illustrate the icon in light tones, designed for simplicity and readability.

#### 5.1.2. General Structure of the SmartRoutes Application Screens

**Figure 20:**
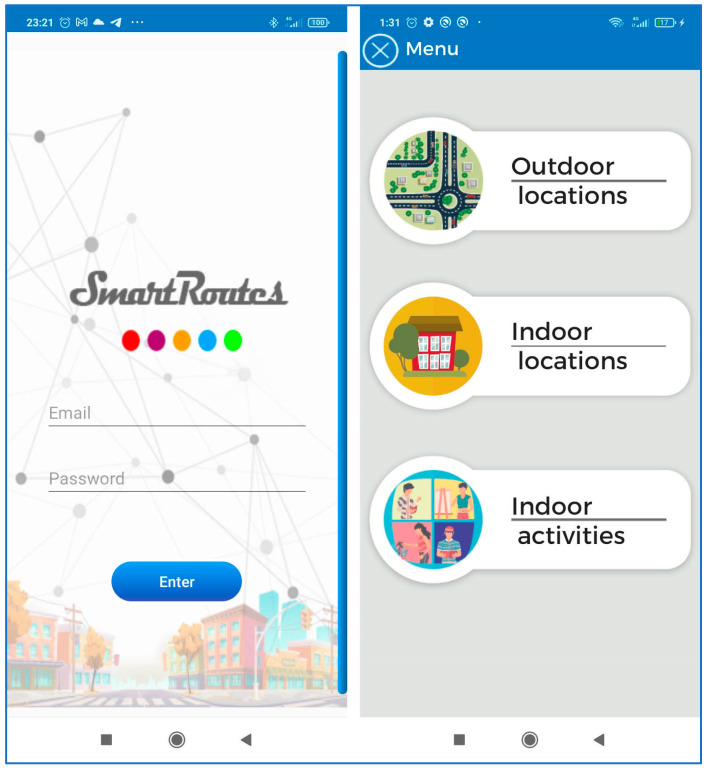
Login screen and menu of the SmartRoutes application.

**Figure 21:**
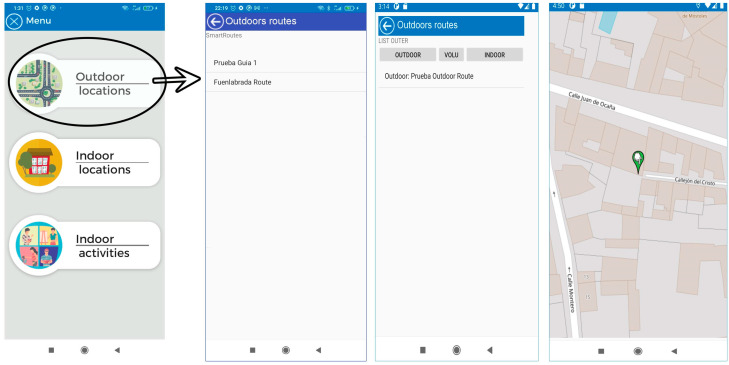
Outdoor location option.

Access to the application is initiated from a login screen. The user registers within the application and then accesses the application’s menu. Figure 20 illustrates this flow. Within this menu, the user can access outdoor location services, which display an OpenStreetMap. The starting point of the location displayed here is determined by information entered from a web platform SmartRoutes Manager. The SmartRoutes application receives this information via a server, as shown in Figure 21, which details this process. It is worth noting that the SmartRoutes Manager web platform has undergone extensive code optimization, improving and simplifying the data structure in an efficient manner to enhance response times and communication with the server [27].

**Figure 22:**
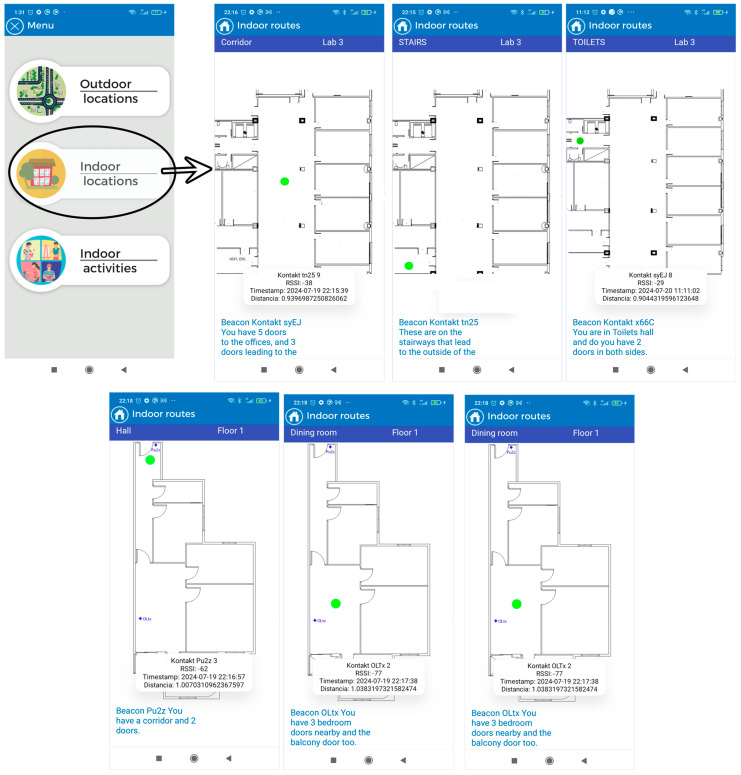
Indoor location option at a specific building in the university.

The indoor location option provides a screen displaying the building map for user guidance and positioning using Bluetooth beacon technology. The application shows the name of the location and the floor or building (e.g., Floor 1, 2, Building A, B, etc.) in two text fields at the top of the screen. Additionally, at the bottom of the screen, another text field displays a description of the location. All text fields are read aloud by a voice prompt to inform the user both visually and audibly about their current location. The central part of the screen displays the location map, which updates according to the user’s position and provides relevant information about the location. The application continuously scans in cycles of 1 s to keep the position constantly updated. To optimize storage on the device, the temporary table and other tables, except for the user table, are deleted once the application is closed, as shown in Figure 22.

**Figure 23:**
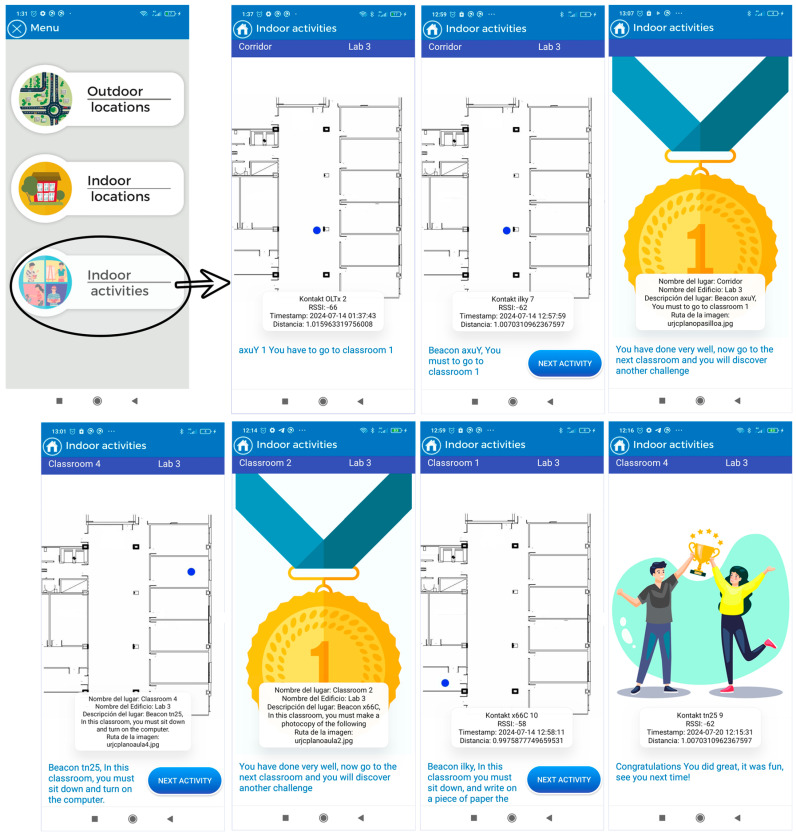
Indoor activities option.

Finally, the user has the option to engage in a series of indoor cognitive activities designed with gamification elements. This option allows the user to locate and navigate through different classrooms. When the user reaches a classroom, the system indicates their location and proposes an activity to be performed there. After 10 min, a “next activity” button appears on the application, which, when pressed, provides feedback to the user according to the activity and encourages them to discover new tasks at that location. Once all activities are completed, the application congratulates the user for completing all challenges in the classrooms and bids them farewell. Figure 23 illustrates this operational flow.

**Figure 24:**
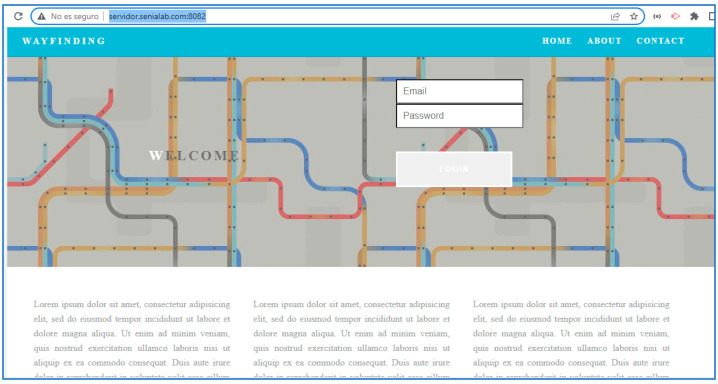
SmartRoutes Manager or SmartRM web platform for select outdoor locations.

The homepage of the SmartRoutes Manager web platform is shown in Figure 24.

**Figure 25:**
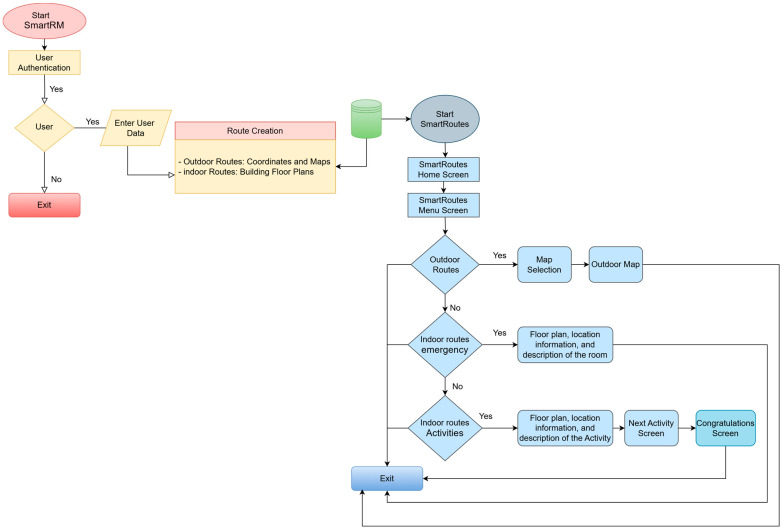
Flowchart of SmartRM and SmartRoutes.

The Figure 25 shows the flowchart of the guidance system composed of the SmartRM web platform and the SmartRoutes mobile application.

## 6. Discussion

Throughout this article, we have analyzed how although in previous years there was no literature focused on guiding people with cognitive disabilities, some research teams have begun to explore this field, particularly in relation to indoor navigation for this group. An example of this is the work of Lancioni et al. [25], who developed a tool for guiding individuals with severe visual and cognitive disabilities in indoor environments. Similarly, the project by Dees and Dirks et al. [23], as previously mentioned, examines the potential of smartphone-based indoor navigation applications to improve the independence of people with cognitive disabilities. Additionally, Zhou et al. [26] propose a solution focused on the group of people providing medical assistance during emergency evacuations, an area that previously lacked studies. However, none of these projects offer solutions tailored to the specific psychological and physical needs of people with mild to moderate cognitive disabilities, such as those with Down syndrome or other cognitive syndromes, to help them navigate indoor environments and cope with challenging emergency situations or daily tasks. In contrast, our project develops an accessible, usable, and tailored tool for the specific needs of this group, providing a solution that enables them to handle high-stress situations, such as emergencies in hostile environments, as well as everyday situations.

The SmartRoutes system enhances existing navigation systems by focusing on cognitive accessibility, overcoming limitations seen in other solutions, which have relied on Wi-Fi and Li-Fi technologies, which are predominantly designed for mobility-impaired users. In contrast, SmartRoutes is engineered for individuals with mild to moderate cognitive disabilities, emphasizing intuitive, voice-guided assistance. While Pilski et al. [21] applied beacon technology for users with hearing impairments, SmartRoutes optimizes beacon-based indoor and outdoor navigation to aid cognitively disabled individuals. The integration of real-time positioning and emergency evacuation support is where SmartRoutes particularly excels, as existing systems generally lack comprehensive crisis handling.

Additionally, Excursiona [28] is an outdoor, group-based collaboration platform designed for coordinated navigation and interaction during excursions. Although it supports emergency notifications, SmartRoutes provides more targeted, individual safety features specifically for cognitively impaired users navigating complex spaces in emergency situations. Table 15 compares the main state-of-the-art applications relevant to our research.

This comparison highlights the unique strengths of SmartRoutes, particularly in emergency navigation and cognitively accessible guidance. Excursiona stands out for its group-based collaboration capabilities; however, it lacks the specialized focus on emergencies and cognitive disabilities that SmartRoutes provides. Accordingly, none of these projects offer solutions tailored to the specific psychological and physical needs of individuals with mild to moderate cognitive disabilities, such as those with Down syndrome or other cognitive syndromes, to support them in navigating indoor environments and addressing complex emergency situations or daily tasks. In contrast, our project develops an accessible, usable, and customized tool specifically designed for this group’s needs, providing a solution that enables them to manage high-stress situations such as emergencies in challenging environments as well as everyday scenarios.

Additionally, this comparison highlights the differences in positioning accuracy among the systems, a critical factor in guiding individuals with cognitive disabilities. SmartRoutes, leveraging Bluetooth Low Energy (BLE) and beacon technology, offers high-precision real-time positioning in indoor environments, which is essential for guiding users to emergency exits with minimal error. In contrast, systems like Excursiona rely primarily on outdoor GPS, which lacks the granularity required for indoor navigation and precise emergency guidance. Chen’s application, though effective for general mobility, depends on static instructions via Wi-Fi and Li-Fi, limiting its adaptability to changes in the user’s surroundings. Pilski et al. utilize auditory guidance through beacons, though without the level of accuracy achieved by SmartRoutes in dynamic, high-stakes scenarios.

With its high-precision technology, SmartRoutes offers a significant advantage, particularly for safely and confidently guiding cognitively diverse users in complex and stressful indoor environments.

We acknowledge that the current 20 s update interval, in the context of emergencies, may not align with the rapid localization capabilities offered by some state-of-the-art systems. However, our system is specifically designed for users with cognitive disabilities, for whom simplicity and clarity are essential. The slower update rate ensures that instructions remain clear and easy to process, thereby reducing the likelihood of confusion during stressful situations, such as emergencies. Additionally, our system operates effectively even in the absence of network connectivity, providing reliable guidance during critical moments. The platform facilitates the creation of user-tailored routes and content, while the mobile application retrieves and presents these routes, offering intuitive, real-time guidance. This modular design effectively supports users’ navigation in both routine and emergency scenarios. Future enhancements may focus on optimizing the update frequency to improve responsiveness while maintaining ease of use for the target user group.

In future use cases of the solution comprising the SmartRM web platform and the SmartRoutes mobile platform, the solution could be adapted for use on a smartwatch. This would enable guiding users in hostile environments or other circumstances through acoustic alerts, using short and concise messages that are cognitively understandable, thereby overcoming the physical and psychological obstacles faced by individuals with cognitive disabilities, such as those with Down syndrome, Prader–Willi syndrome, Williams syndrome, or Fragile X syndrome.

## Figures and Tables

**Table 1:** Most commonly used technologies for people with Down syndrome.

Educational Support Technologies for People with Down Syndrome				
Augmentative and Alternative Communication (AAC) systems: non-verbal communication, such as voice boards, sign languages, facial expressions, gestures, etc.	Augmented reality	Navigation and location systems using beacons	Educational robots and simulators	Educational video games that include gamification and interaction

**Table 2:** Measurements between beacon and device.

Distance (m)	RSSI (dBm)
0.25	−47
0.50	−54
1	−62
2	−70
3	−72
4	−74
5	−78
6	−80
7	−83
8	−86
9	−88
10	−91
11	−94
12	−96

**Table 3:** Beacon to device ratio calculation table.

Distance (m)	RSSI (dBm)	Reference Value (Ratio)
0.25	−47	0.796610169
0.50	−54	0.915254237
1	−62	1.050847458
2	−70	1.186440678
3	−72	1.220338983
4	−74	1.254237288
5	−78	1.322033898
6	−80	1.355932203
7	−83	1.406779661
8	−86	1.457627119
9	−88	1.491525424
10	−91	1.542372881
11	−94	1.593220339
12	−96	1.627118644

**Table 4:** Table of *x* and *y* values between beacon and device.

*x*	*y*
0.25	0.796610169
0.50	0.915254237
1	1.050847458
2	1.186440678
3	1.220338983
4	1.254237288
5	1.322033898
6	1.355932203
7	1.406779661
8	1.457627119
9	1.491525424
10	1.542372881
11	1.593220339
12	1.627118644

**Table 5:** Calculation of predictive distance, with coordinates *a* and *b*, between beacon and device.

RSSI (dBm)	Current Distance (m)	Ratio	Predictive Distance
−47	0.25	0.796610169	0.983419543
−54	0.50	0.915254237	1.007339944
−62	1	1.050847458	1.031719637
−70	2	1.186440678	1.053622815
−72	3	1.220338983	1.058773226
−74	4	1.254237288	1.063806663
−78	5	1.322033898	1.073545043
−80	6	1.355932203	1.078260193
−83	7	1.406779661	1.085153344
−86	8	1.457627119	1.091843461
−88	9	1.491525424	1.096197093
−91	10	1.542372881	1.102576573
−94	11	1.593220339	1.108784451
−96	12	1.627118644	1.112832616

**Table 6:** *c* coordinate predictive distance calculation between beacon and device.

RSSI (dBm)	Current Distance (m)	Ratio	Predictive Distance
−47	0.25	0.796610169	0.951699906
−54	0.50	0.915254237	0.975620306
−62	1	1.050847458	1
−70	2	1.186440678	1.021903177
−72	3	1.220338983	1.027053589
−74	4	1.254237288	1.032087025
−78	5	1.,322033898	1.041825406
−80	6	1.355932203	1.046540555
−83	7	1.406779661	1.053433707
−86	8	1.457627119	1.060123823
−88	9	1.491525424	1.064477455
−91	10	1.542372881	1.070856935
−94	11	1.593220339	1.077064813
−96	12	1.627118644	1.081112978

**Table 7:** Cartesian coordinates of trilateration beacons in a specific building.

Beacons	References	*x* Coordinate	*y* Coordinate
Kontakt	*a*	0	0
Kontakt 2	*b*	39	34
Kontakt 3	*c*	57	0

**Table 8:** Emergency and activities cases, time response of application testing.

**User Location Times**		
**Corridor**	**Dining Room**	**Room**
20 s	20 s	20 s
**User Location Times in each activity**		
**Activity 1 (Corridor)**	**Activity 2 (Dining Room)**	**Activity 3 (Room Activity)**
30 s	30 s	30 s

**Table 9:** Emergency case in a residential building.

Emergencies Indication in Each Location’s Points		
Corridor	Dining Room	Room
This is the access area to the house; from here, the user needs to go to the dining room.	In this area, the user is informed about the doors and access to other rooms, the windows, and the situation of the balcony, and is given a description of the room’s layout, furniture, and fixed elements that can be found in the dining room.	In this area, the user is provided with a description of the room’s layout, including the placement of the window, furniture, and fixed elements that can be found in the room.

**Table 10:** Functional activities case in a residential building.

Activity Description in Each Location’s Points		
Activity 1 (Corridor)	Activity 2 (Dining Room)	Activity 3 (Room Activity)
This is the access area to the house; from here, the user must proceed to the dining room.	In this room the user must turn on the TV and select a thematic channel and say out loud which program is being broadcasted.	In this room, the user finds a window and several pieces of furniture, among them a table, a PC, and a printer; the user must sit down and write a text on the PC and print it.

**Table 11:** Activities case, time response of application testing.

User Location Times in Each Classroom Or Activity				
Hall Activity 1	Stairs Area Activity 2	Toilet Area Activity 3	Classroom Activity 4	Hall Activity 5
30 s	30 s	30 s	30 s	30 s

**Table 12:** Emergency case in a building at the university.

User Location Times		
Hall	Stairs Area	Toilet Area
20 s	20 s	20 s

**Table 13:** Emergency case in a building at the university.

Emergency Indication in Each Location’s Points		
Hall	Stairs Area	Toilet Area
Beacons Kontakt axuY, Kontakt ilky, and Kontakt syEJ. You have 5 doors to the offices, and 3 doors leading to the lobbies.	Beacon Kontakt tn25. These are on the stairways that lead to the outside of the building.	Beacon Kontakt x66C. You are in toilets hall.

**Table 14:** Case of functional activities in a building at the university.

Activity Description in Each Location’s Points				
Hall Activity 1	Stairs Area Activity 2 Classroom 1	Toilet Area Activity 3 Classroom 2	Classroom Activity 4 Classroom 4	Hall Activity 5 Classroom 3
Beacon axuY. You must go to classroom 1.	Beacon ilky. In this classroom, you must sit down and write on a piece of paper the name of your favorite movie, say its name out loud, and why you like it.	Beacon x66C. In this classroom, you must make a photocopy of the following.	Beacon tn25. In this classroom, you must sit down and turn on the computer.	Beacon syEJ. In this classroom, you must write on the board what your favorite song is and why you like it.

**Table:** Functional activities case in a building at the university.

Comparison Among Wayfinding Applications for Cognitively Disabled Users				
Feature	SmartRoutes	Excursiona [28]	Chen [16]	Pilski et al. [20]
Target Audience	Individuals with cognitive disabilities	Group excursions (children, firefighters, etc.)	Mobility-impaired users	Hearing disabilities
Collaboration Features	Indoor/outdoor route guidance, emergency exits	Map-based collaboration, chat, point sharing	Wi-Fi and Li-Fi for navigation	Beacon technology for auditory guidance
Real-Time Positioning	Yes, for indoor and outdoor environments	Yes, with user avatars and group positioning	No, relies on static instructions	Yes, beacon technology
Technological Base	Bluetooth, iBeacon, BLE, Android	Flutter, Firebase, Google Maps	Wi-Fi, Li-Fi	Bluetooth, beacons
Cross-Platform Support	Primarily Android	Cross-platform (iOS and Android) via Flutter	Android/iOS (limited by device compatibility)	Android/iOS
Emergency Handling	Yes, real-time guidance to safe exits	Yes, with emergency notifications	No emergency handling	No emergency handling
Specific Use Case 30 s	Navigating complex spaces and emergencies	Group-based outdoor excursions	Indoor mobility navigation	Indoor auditory navigation using beacons

## Data Availability

Data are contained within the article.

## References

[1] European Commission Communication from the Commission to the European Parliament, the Council, the European Economic and Social Committee, the Committee of the Regions on the Road to Automated Mobility: An EU Strategy for Mobility of the Future. https://eur-lex.europa.eu/legal-content/EN/TXT/?uri=CELEX%3A52018DC0283.

[2] DGT Directorate General for Traffic, Ministry of the Interior of the Government of Spain. https://www.dgt.es/muevete-con-seguridad/tecnologia-e-innovacion-en-carretera/Dispositivos-de-presenalizacion-V16/.

[3] European Commission An EGNSS Application Providing an End-to-End Solution Based on the SAR/Galileo Service and Particularly Using the Return-Link-Message (RLM), to Improve the Mobility and Safety of Citizens. https://cordis.europa.eu/article/id/231914-improved-search-and-rescue-with-wristworn-personal-locator-beacons/es/.

[4] Baejah, Ahmadi N., Adiono T. Indoor Localization System Based on Bluetooth Low Energy Beacons and Spiking Neural Networks. Proceedings of the 2023 IEEE 66th International Midwest Symposium on Circuits and Systems (MWSCAS).

[5] Sarcevic P., Csík D., Pesti R., Stefanoni M., Sárosi J., Odry Á. Fingerprint-based fusion of magnetic field data with multiple wireless technologies for indoor mobile robot positioning. Proceedings of the 2023 13th International Conference on Indoor Positioning and Indoor Navigation (IPIN).

[6] Srinithi P., Kalpanadevi S., Rekha P., Divya N., Rajkumar M., Aathiba D. A Novel Paradigm of Indoor Navigation System using Li-Fi Technology. Proceedings of the 2nd International Conference on Automation, Computing and Renewable Systems (ICACRS).

[7] Pei F., Shi M., Kong X. Multi-Level Feature Extraction and Autoregressive Prediction Based Wi-Fi Indoor Fingerprint Localization. Proceedings of the 2023 China Automation Congress (CAC).

[8] Wang J., Sun S., Ning Y., Zhang M., Pang W. Ultrasonic TDoA Indoor Localization Based on Piezo electric Micromachined Ultrasonic Transducers. Proceedings of the 2021 IEEE International Ultrasonics Symposium (IUS).

[9] Cheng B., Huang Y., Zou C. (2024). Robust Indoor Positioning with Smartphone by Utilizing Encoded Chirp Acoustic Signal. Sensors.

[10] United Nations Days of Persons with Disabilities. https://www.un.org/en/observances/day-of-persons-with-disabilities/background.

[11] ALAS Latin American Association of Sociology, Intellectual Disability: An Interpretation Within the Framework of the Social Model of Disability. https://www.redalyc.org/journal/5886/588662103007/html/.

[12] Malachowski M. (2016). Understanding Mental Disorders: Your Guide to DSM-5, by the American Psychiatric Association: Washington, DC: American Psychiatric Association, 2015. 388p. ISBN 978-1-58562-491-1. $24.95 (softcover). Med Ref. Serv. Q..

[13] Kuo H.J., Sung C., Newbutt N., Politis Y., Robb N., Brooks A.L., Brahman S., Kapralos B., Nakajima A., Tyerman J., Jain L.C. (2021). Current Trends in Technology and Wellness for People with Disabilities: An Analysis of Benefit and Risk. Recent Advances in Technologies for Inclusive Well-Being.

[14] United Nations World Down Syndrome Day 21 March. https://www.un.org/en/observances/down-syndrome-day.

[15] Krasniqi V., Zdravkova K., Dalipi F. (2022). Impact of Assistive Technologies to Inclusive Education and Independent Life of Down Syndrome Persons: A Systematic Literature Review and Research Agenda. Sustainability.

[16] Chen I. (2024). Accessible Navigation Mapping: Supporting People with Mobility Disabilities for Wayfinding; Evaluating Campus Infrastructure: Assessing Prioritization of Disabled Communities in Universities Through Architecture. Bachelor’s Thesis.

[17] Chen J., Shi T., Li N. (2021). Pedestrian evacuation simulation in indoor emergency situations: Approaches, models and tools. Saf. Sci..

[18] Valizadeh M., Ranjgar B., Niccolai A., Hosseini H., Rezaee S., Hakimpour F. (2024). Indoor augmented reality (AR) pedestrian navigation for emergency evacuation based on BIM and GIS. Heliyon.

[19] Giudice N.A., Guenther B.A., Kaplan T.M., Anderson S.M., Knuesel R.J., Cioffi J.F. (2020). Use of an Indoor Navigation System by Sighted and Blind Travelers: Performance Similarities across Visual Status and Age. ACM Trans. Access. Comput..

[20] Pilski M., Mikułowski D., Terlikowski G. (2023). An indoor campus navigation system for users with disabilities. Studia Informatica. Stud. Informatica. Syst. Inf. Technol..

[21] Dees J., Dirks S. (2023). Cognitive Accessibility of Indoor Navigation Apps. Stud. Health Technol. Inform..

[22] Lancioni G.E., O’reilly M.F., Sigafoos J., Desideri L., Alberti G., Chiariello V., Nozzolillo A. (2020). Smartphone-Based Technology to Help Individuals with Intellectual Disability and Blindness Manage Basic Indoor Travel. Adv. Neurodev. Disord..

[23] García-Catalá M.T., Rodríguez-Sánchez M.C., Martín-Barroso E. (2020). Survey of indoor location technologies and wayfinding systems for users with cognitive disabilities in emergencies. Behav. Inf. Technol..

[24] Zhou T., Xia P., Zhu Q., Du J. (2023). Cognition-driven navigation assistive system for emergency indoor wayfinding (CogDNA): Proof of concept and evidence. Saf. Sci..

[25] Yoo S.-J., Choi S.-H. (2022). Indoor AR Navigation and Emergency Evacuation System Based on Machine Learning and IoT Technologies. IEEE Internet Things J..

[26] Özbeşer H., Tüzün E.H., Dericioğlu B., Övgün Ç.D. (2024). Effects of Cognitive Orientation to Daily Occupational Performance and Conductive Education Treatment Approaches on Fine Motor Skills, Activity and Participation Limitations in Children with Down Syndrome: A Randomised Controlled Trial. J. Autism Dev. Disord..

[27] Steve S. (2009). Even Faster Web Sites: Performance Best Practices for Web Developers.

[28] Ortega Cordovilla M., Garrido Merino S., Bravo Santos C., Molina Díaz A.I., Ortega Cantero M. (2024). Excursiona: Enhancing collaborative and awareness-driven navigation for outdoor group excursions. SoftwareX.

